# Transcriptional Characterization of Stage I Epithelial Ovarian Cancer: A Multicentric Study

**DOI:** 10.3390/cells8121554

**Published:** 2019-12-01

**Authors:** Enrica Calura, Matteo Ciciani, Andrea Sambugaro, Lara Paracchini, Giuseppe Benvenuto, Salvatore Milite, Paolo Martini, Luca Beltrame, Flaminia Zane, Robert Fruscio, Martina Delle Marchette, Fulvio Borella, Germana Tognon, Antonella Ravaggi, Dionyssios Katsaros, Eliana Bignotti, Franco Odicino, Maurizio D’Incalci, Sergio Marchini, Chiara Romualdi

**Affiliations:** 1 Department of Biology, University of Padova, 35121 Padua, Italy; enrica.calura@unipd.it (E.C.); sambu96@gmail.com (A.S.); giuseppe.benvenuto@phd.unipd.it (G.B.); militesalvatore@gmail.com (S.M.); paolo.martini@unipd.it (P.M.); chiara.romualdi@unipd.it (C.R.); 2 Department of Cellular, Computational and Integrative Biology—CIBIO, University of Trento, 38123 Povo Trento, Italy; matteo.ciciani@gmail.com; 3 Department of Oncology, Istituto di Ricerche Farmacologiche Mario Negri IRCCS, 20156 Milano, Italy; lara.paracchini@marionegri.it (L.P.); luca.beltrame@marionegri.it (L.B.); sergio.marchini@marionegri.it (S.M.); 4 Unit of Biological Adaptation and Ageing UMR8256, Institute of Biology Paris-Seine, Sorbonne University, 75005 Paris, France; flaminia.zane@gmail.com; 5 Clinic of Obstetrics and Gynaecology, University of Milano-Bicocca, San Gerardo Hospital, 20900 Monza, Italy; robert.fruscio@unimib.it (R.F.); m.dellemarchette@gmail.com (M.D.M.); 6 Department of Surgical Science and Gynecology, Azienda Ospedaliero Universitaria, Città della Salute, presidio S.Anna, University of Torino, 10126 Torino, Italy; fulvio.borella87@gmail.com (F.B.); d.katsaros@libero.it (D.K.); 7 Division of Obstetrics and Gynecology, ASST Spedali Civili di Brescia, 25123 Brescia, Italy; germanatognon@gmail.com (G.T.); bignottieliana@gmail.com (E.B.); franco.odicino@gmail.com (F.O.); 8 Angelo Nocivelli Institute of Molecular Medicine, University of Brescia and ASST-Spedali Civili of Brescia, 25123 Brescia, Italy; antonella.ravaggi@unibs.it; 9 Department of Clinical and Experimental Sciences, Division of Obstetrics and Gynecology, University of Brescia, 25123 Brescia, Italy

**Keywords:** stage I ovarian cancer, cancer histotypes, pathways, gene expressions

## Abstract

Stage I epithelial ovarian cancer (EOC) represents about 10% of all EOCs. It is characterized by a complex histopathological and molecular heterogeneity, and it is composed of five main histological subtypes (mucinous, endometrioid, clear cell and high, and low grade serous), which have peculiar genetic, molecular, and clinical characteristics. As it occurs less frequently than advanced-stage EOC, its molecular features have not been thoroughly investigated. In this study, using in silico approaches and gene expression data, on a multicentric cohort composed of 208 snap-frozen tumor biopsies, we explored the subtype-specific molecular alterations that regulate tumor aggressiveness in stage I EOC. We found that single genes rather than pathways are responsible for histotype specificities and that a cAMP-PKA-CREB1 signaling axis seems to play a central role in histotype differentiation. Moreover, our results indicate that immune response seems to be, at least in part, involved in histotype differences, as a higher immune-reactive behavior of serous and mucinous samples was observed with respect to other histotypes.

## 1. Introduction

Epithelial ovarian cancer (EOC) is a highly lethal and heterogeneous gynecological disease. It is characterized by a complex histopathological and molecular heterogeneity, and it is composed of five main histological subtypes (mucinous, endometrioid, low and high grade serous, and clear cell), which have peculiar genetic lesions, different biomarkers, response, and outcomes to standard treatments [1,2,3].

Studies have provided evidence that most ovarian cancers arise from tissues that are not normally present in the ovary such as fallopian tubes, endometrial tissue, or tubal-mesothelial junction, thus determining differences among the various histologic types [4,5,6,7].

In recent years, many efforts have been dedicated to provide a catalogue of molecular alterations in advanced EOC with the goals of developing and deploying therapies to improve patient survival. Indeed, transcriptome alterations are the proxy of tumor molecular activities and their investigations may suggest which strategies the tumor cells adopt to escape immune system recognition and death, to proliferate and to spread.

Currently, a large amount of data are available on advanced EOC, especially on the high-grade serous histotype [8], but, given its infrequent incidence, few studies were dedicated to the study of stage I EOC, when the tumor is confined to the ovaries or fallopian tubes.

Moreover, very recently, tumor microenvironment composition, tumor-immune cell interaction, and the association genotypes-immunophenotypes have been recognized as key aspects in cancer progression and have been used successfully for patients’ stratification [9,10]. From the therapeutic point of view, the dissection of these complex interactions will provide the opportunity to identify new biomarkers, to develop novel drugs or therapeutic strategies, as well as to dissect novel mechanistic insights. If immune-checkpoint inhibitors alone have demonstrated to achieve only a small improvement in late stage EOC patients’ prognosis [11], the combination of immunotherapy agents and other biologic therapies may prove to be significantly more effective. Noteworthy, these issues have not been explored yet in stage I EOC.

In the last years, using transcriptome data, our group addressed two complementary issues on stage I EOC: (i) the identification of a prognostic signature shared across the different histotypes, and (ii) the identification of histotype-specific biomarkers.

Regarding the first aim, using a pathway-based integrative method [12] on an Italian multicentric cohort of stage I EOC patients, we identified a prognostic regulatory network composed of genes and miRNAs wiring cell cycle, Activins and Hedgehog signaling pathways [13]. Using the expression levels of the elements of the network, we proposed a score to stratify patients into different classes of risk with a higher classification performance than conventional clinical/pathological classifiers [13]. Among the network elements, we confirmed the key prognostic role of mir-200c previously found by our team [14]. Not only short but also two long non-coding RNA (PVT1 and lnc-SOX4-1) were found to be prognostic biomarkers in stage I EOC playing a role in tumor aggressiveness through the activation of TGF*β* and PIK3/AKT pathways [15].

Regarding the second aim, we identified small but robust miRNA signatures for clear cell (miR-30a and miR-30a*) and mucinous (miR-192 and miR-194) subtypes [16] showing that, through an miR-192/miR-194 loop, mucinous samples interact with the prognostic circuit through the Activins pathway [17], suggesting a histotype-specific mechanism of the prognostic signature modulation.

Thus, our recent studies suggest that some mechanisms, possibly related to the genetic patient background or to the common anatomical site of growth, are playing a key role in driving tumor prognosis and that the different molecular specificities of the different histotypes act as satellite and synergic events in modulating tumor aggressiveness and relapse.

With this hypothesis, using in silico approaches and gene expression data, we tried to describe the subtypes-specific molecular alterations that modulate the intrinsic mechanisms that regulate tumor aggressiveness in stage I EOC. We analyzed the transcriptome of a large multicentric cohort of stage I EOC patient and provide a global overview of the transcriptome alterations focusing on different questions: (i) known signatures proposed for high-grade serous ovarian carcinoma by The Cancer Genome Atlas (TCGA) consortium [8] can be used to explain stage I EOC heterogeneity? (ii) deconvolution strategies to evaluate the presence of immune-infiltrates can elicit different histotype-specific immune responses? (iii) can we identify the biological pathways along with their connections that characterize the different histotypes?

## 2. Results and Discussion

### 2.1. Patient Cohorts and Sample Collection

We retrospectively collected tumor samples from patients diagnosed at stage I EOC from three independent Italian cohorts, for a total of 208 snap-frozen tumor biopsies. (i) 135 samples were collected at Gynecology Department of San Gerardo Hospital (Monza, Italy) and conserved in the tissue bank of the Department of Oncology of IRCCS Mario Negri Institute (Milano, Italy), (ii) 26 samples at the A. Nocivelli Institute for Molecular Medicine, Division of Obstetrics and Gynecology (ASST Spedali Civili of Brescia, University of Brescia, Brescia, Italy) and (iii) 47 samples at the Department of Gynecology-Oncology (University of Torino, Torino, Italy). Primary tumor tissues were collected at the time of diagnosis and first surgery prior to any chemotherapy treatment. Samples were submitted for a complete staging procedure according to the International Federation of Gynecological and Obstetrics (FIGO) criteria with a diagnosis of stage I EOC. Tumor histological types were determined following World Health Organization (WHO) standards.

Samples were randomly stratified into a training set and validation set to have sizable histotype groups inside each set; 76 samples of the Milano cohort were dedicated to the training set and 59 samples of Milano together with Brescia and Torino cohorts for the validation set. Histopathological and clinical annotations are described in Table 1. The training set (*n* = 76) included 16 clear cell (Cc), 19 endometroid (End), 17 mucinous (Muc) and 16 high grade serous (SerHigh) and eight low grade serous histotypes, while the validation sets (*n* = 132) were 22 Cc, 55 End, 21 Muc, 26 SerHigh, and 8 SerLow histotypes. The training set underwent genome-wide characterisation, while the validation set was used for RT-qPCR experiments.

Training and validation sets have an average follow-up of nine and six years respectively, comparable mean age at the time of diagnosis, similar percentage of relapse, and comparable histo-pathological and clinical characteristics, such as grade and FIGO substage.

### 2.2. Stage I Classification Using Advanced Ovarian Cancer Expression Subtypes

Based on gene expression profiles, Tothill et al. [18] identified six subtypes of invasive high grade EOC displaying distinct levels and patterns of immune cell infiltration with prognostic implications. The Cancer Genome Atlas (TCGA) subsequently revised these transcriptional subtypes and identified four classes [8]: differentiated subtype (DIF) characterized by gene expression profile of well differentiated and low malignant potential cancers; mesenchymal subtype (MES) with high stromal content; immunoreactive subtype (IMR) which contains most of the immune transcripts associated with tumor inflammatory response and proliferative subtype (PRO) characterized by the expression of genes associated with cell division. Although molecularly different, these classes of patients were not found to be different in survival.

**Figure 1:**
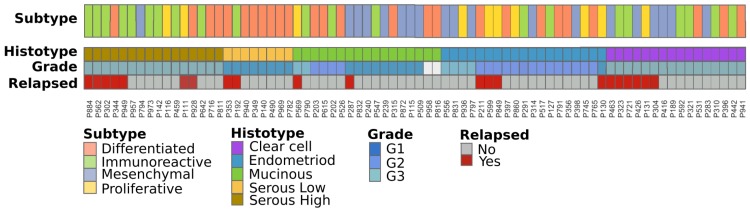
Subtypes classification of stage I Epithelial ovarian cancer (EOC) samples as defined by Tothill et al. along with their clinical information: (i) immunoreactive (green)—associated with infiltration of immune cells, (ii) proliferative (yellow)—a low stromal expression subtype with high levels of circulating CA125, (iii) differentiated (orange)—a poor prognosis subtype displaying strong stromal response, correlating with extensive desmoplasia, and (iv) mesenchymal (light blue)—with high expression of N- and P-cadherins.

Here, we wonder whether these signatures can explain, at least in part, the transcriptome variability of stage I EOC samples and if specific histotypes are associated with one or more of these signatures. Figure 1 shows the results of the classification of our samples into the five mentioned subtypes along with their clinical information. All samples can be classified without ambiguity into one of the four classes, obtaining 26 DIF (34%) samples, 19 MES (25%), 21 IMR (28%), and 10 PRO (13%). Although transcriptional subtypes are distributed across all the histotypes, we found a significant association between Tothill’s classification and histotype (*p*-value = 0.002—Supplementary File 1). Specifically, end histotype samples are mainly PRO, MES subtype are mainly Cc and Muc and virtually lacks of Ser samples, which otherwise show a clear difference between high and low grades; we observed that SerHigh are preferentially IMR, and SerLow are almost exclusively DIF.

In agreement with this last result, we observed a significant association with grade (*p*-value = 0.02—Supplementary File 1). As expected, DIF subtypes are less commonly high grade samples compared to low grade as a consequence of the increasing nuclear atypia from low to high grades. We also observed an increasing presence of IMR samples along with the increase of tumor grade, suggesting that a high grade tumor can elicit an immune system response more than lower grade samples.

Taken together, our results suggest a different involvement of the immune system and of the cell signaling pathways (especially those dedicated to cell proliferation, cell dedifferentiation, and growth) in different stage I EOC grades and histotypes. To better explore these hypotheses, we decided to (i) study the immune system involvement of our stage I samples using dedicated computational approaches and (ii) provide a characterization of cell signaling pathways across the different histotypes.

### 2.3. Immuno-Phenotype of Stage I EOC Patients

The levels of infiltrating immune cells in tumors are often associated with growth, cancer progression, and poor patient outcome [19,20]. To understand the immune system contribution to the transcriptome of stage I EOC samples, we used two different approaches: (i) a deconvolution method proposed by Chen et al. [21], and (ii) a score method proposed by Charoentong et al. [9].

**Figure 2:**
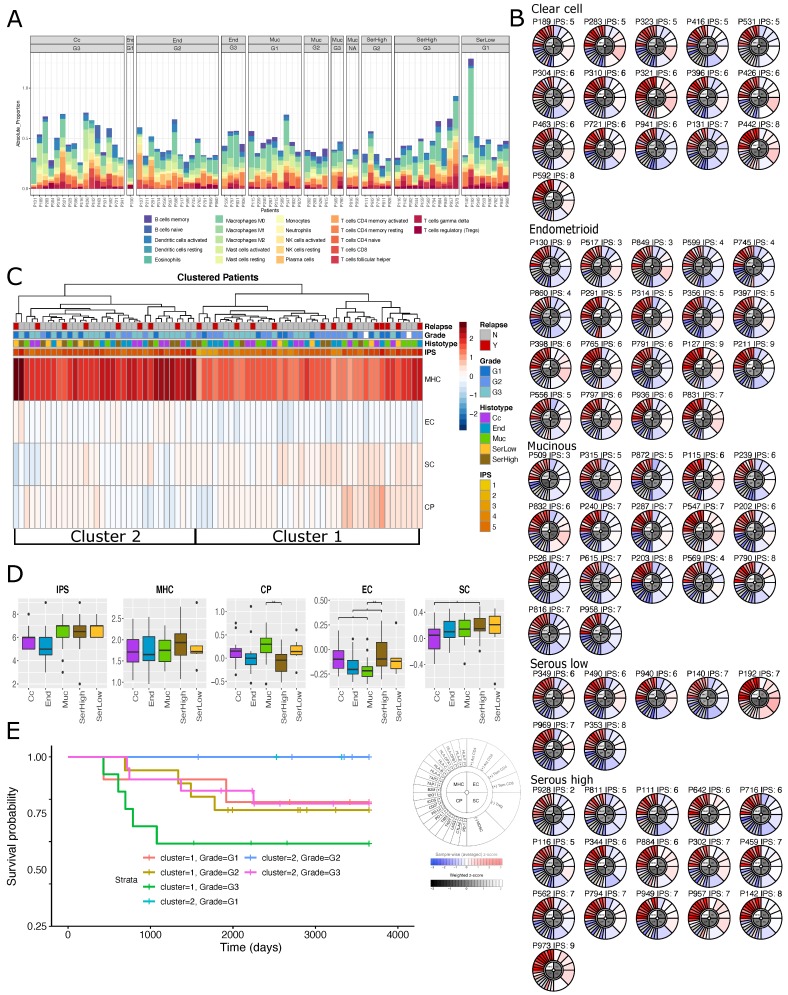
Immune cell composition and behavior in stage I EOC samples. (**A**) absolute fractions of immune cell types per sample obtained using a CIBERSORT deconvolution method; (**B**) patient’s immunophenograms grouped by histotypes and ordered by grade; (**C**) cluster analysis of patients using major histocompatibility complex (MHC), checkpoints immunomodulators (CP), immune effector cells (EC) and suppressor cells (SC) values along with histo-pathological annotations and immunophenoscores (IPS). (**D**) box-plots of IPS along with its four components (MHC, CP, SC and EC) across histotypes and tumor grades. Sample sizes for each boxplot is $n_{Cc}=16,\;n_{End}=19,\;n_{Muc}=17,\;n_{SerHigh}=16,\;n_{SerLow}=7$. (**E**) Kaplan–Meier survival curves of patients grouped according to cluster 1 and 2 (see panel c) stratified by grade.

The deconvolution model by Chen et al. [21] uses gene expression data to estimate and quantify the activation of signatures representative of 22 types of different immune cells. Samples have been divided by histotypes and grades and analyzed separately. Figure 2A shows the estimated proportion of immune cells composition obtained in our samples.

Globally, infiltrated immune cells seem to be a very low fraction of the total amount of profiled cells (an average of 0.5%). Few weak but significant differences in the amount of cell types can be appreciated across histotypes. In particular, NK cells resting are significantly more abundant in Muc (*p* = 0.06), macrophages M2 are significantly more abundant in Cc (*p* = 0.006), while Treg are highly abundant in SerLow (*p* = 0.04) (see Supplementary File 2). Moreover, a higher amount of T cells gamma delta appears high compared to low grades (*p* = 0.01; Supplementary File 2) and a higher amount of NK cells resting are present in low with respect to high grade samples (*p* = 0.02). Finally, abundances of immune cell types do not show significant association with survival (OS or PFS) using multivariate models adjusted for histotypes and grades (Supplementary File 2).

The immunoscore proposed by Charoentong et al. [9] evaluates the expression of immune biomarkers belonging to four categories: (i) antigen processing molecules (MHC), (ii) checkpoints immunomodulators (CP), (iii) immune effector cells (EC) and (iv) suppressor cells (SC). The expression of the biomarkers within these categories are summarized using a z-score called immunophenoscores (IPS) and graphically represented by immunophenogram (IPG). We tested if immunogenicity differences across tumor grades and histotypes can be appreciated using IPG and IPS and if the patient immune system at the diagnosis can have any effect on the disease progression. For detailed description of IPG and IPS, see Methods Section 3.4.

In agreement with the the cell deconvolution approach, all samples, independently by histotype and grade, showed low fractions of infiltrated immune cells. Many genes of the CP class often showed expression values under the detection threshold (gray slices) in both activating and suppressing molecules (Figure 2B).

Considering the global immune-landscape, we found two main clusters of patients characterized by different levels of expression of MHC, EC, SC, and CP expressed biomarkers (Figure 2C). The cluster on the left (hereafter called cluster 2) is moderately enriched in Ser samples (both high and low grade) (63% vs. 37% *p* = 0.07) and interestingly patients in cluster 2 have a significantly longer survival than the other patients when adjusted for Grade (*p* = 0.03, HR = 0.28, CI 95% = 0.08–0.92) (Figure 2E). Going deeper in the comparison across histotypes, we found that the immune checkpoints regulators are highly abundant in Muc and SerLow samples (*p* = 0.01), while effector cells are more abundant in SerHigh histotype and low abundant in Muc (*p* = 0.03) (Supplementary File 3). Moreover, although no significantly different MHC levels can be appreciated across subtypes (Supplementary File 3), we found that cluster 2 has a significantly higher expression of MHC genes with respect to cluster 1 (*p* < 0.001).

MHC genes are highly upregulated compared with the other group of immuno-related genes (Figure 2B,D). High expression of MHC genes in ovarian cancer has been already observed [22] by Gooden and colleagues in a cohort of 270 ovarian cancers, including early and advanced stages, along with tumor infiltrating CD8+ T lymphocytes (CTLs). According to our results, Gooden and colleagues found that HLA-E is highly expressed in virtually all the EOC tumors along with others human leukocyte antigen (HLA) molecules of both class I and II, suggesting that these tumors are characterized by an intact antigen processing apparatus and by abundant CTL infiltration.

Moreover, although not significant, we observed a moderate increase of IPS levels for Muc and Ser samples (both high and low grades) compared to Cc and End (Figure 2C).

Finally, as expected, different grades have slightly different values of IPS (Supplementary File 3). This seems to be mainly due to differences in expression levels of specific immune cells e.g., EC and SC classes dedicated to respectively to effector and suppressor cells show significant differences between high and low grade (activated CD8+ T cells and CD4+ T cells, Tem CD8+ and Tem CD4+ cells: G3 vs. G2 *p* = 0.02; Tregs and Myeloid-derived suppressor cells (MDSCs); G3 vs. G2 *p* = 0.08 Supplementary File 3). Specifically, samples showing the most severe nuclear atypia and the worst prognosis have higher levels of EC and lower level of SC, suggesting that the activity of both these tumor infiltrated T cells might cause a differential tumor visibility to the immune system [23]. Finally, we do not find any association between IPS and survival (Supplementary File 3).

In conclusion, both approaches indicate that Ser samples seem to be more immuno-reactive with respect to other subtypes having activated effector cells, small abundances of NK cell resting, and altered checkpoints.

### 2.4. Transcriptional Alterations of Ovarian Cancer Stage I

To highlight subtype-specific transcriptional alterations, we identified differentially expressed genes across histotypes and performed a Gene Ontology (GO) enrichment analyses (Supplementary File 6). However, given the small number of differentially expressed genes (using *FDR* < 0.05 as threshold), not all the subtype comparisons show significant results; in particular, the analysis using Molecular Function categories gives poor results (Supplementary File 6). On the other hand, using Biological Processes, we found that SerHigh seems to be the most different subtype with respect to the others. Specifically, high and low grade Serous differ for expression in genes involved in cell cycle regulation and DNA repair, while SerHigh and End for genes involved in development, cell–cell communication, migration and Wnt signaling. Finally, SerHigh and Cc show expression differences for genes involved in extracellular structure organization metabolic and catabolic processes (Supplementary File 6). As expected, our results indicate SerHigh as the subtype with a more aggressive phenotype in terms of proliferation, deficient DNA repair, and migration.

The GO functional analyses reported above give a general description of the biological processes characterizing subtypes without taking into account (i) the links among these processes and (ii) the impact that the non-coding part of the transcriptome has on expression alterations. To fill these gaps, we compared integrated transcriptomes (mRNA and miRNA) using a combined network-based approach as described in Materials and Methods. Briefly, each histotype is compared with all the others, and the results are combined into a unified network in which our previously identified prognostic circuit [13] was integrated.

**Figure 3:**
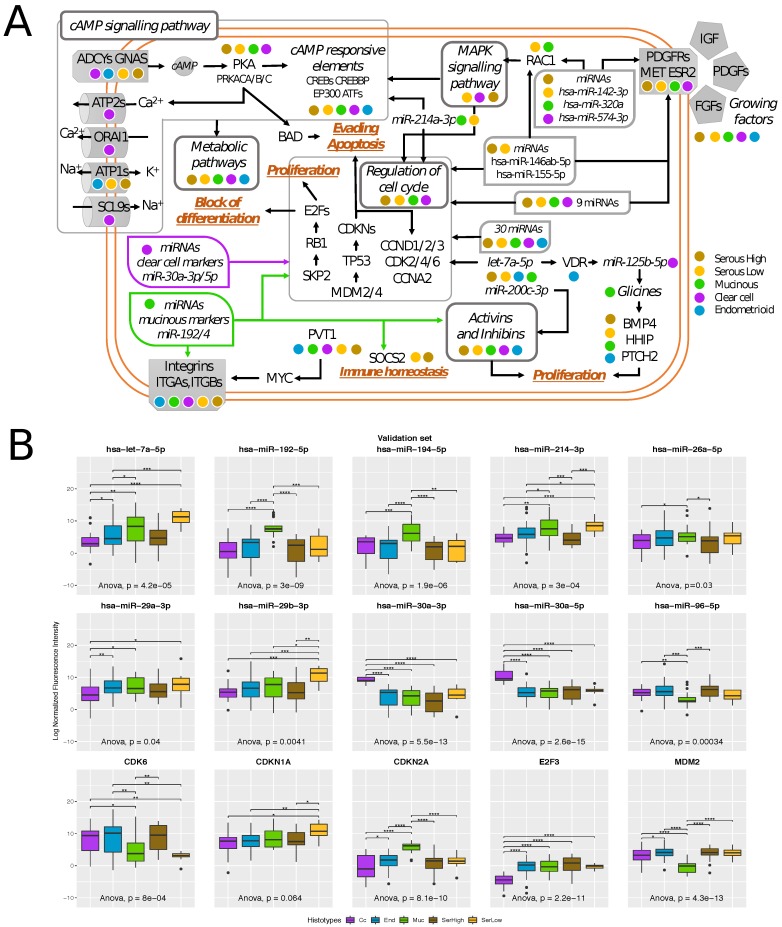
Pathways involved in stage I EOC histotype specificity (**A**) schematic overview of histotype pathway analyses’ results. A complete network is detailed in Supplementary Material 4. Colored points reflect the histotype involvement as described in the legend; (**B**) box-plot of qRT-PCR expression values of selected elements of the network in the training and in the validation set.

The outcome of our analysis is a network composed of 166 genes and 70 miRNAs broadly covering around 13 pathways (Supplementary Material 4). The network has been summarized in Figure 3A, which represents a detailed and comprehensive picture of all the biological processes differentiating EOC histotypes.

The first result that emerges from the structure of the network is that transcriptional differences across histotypes are found at the coding gene level, while at the pathway level all histotypes showed a high degree of overlap. This indicates that every histotype shows alterations on specific coding transcripts and gene-family members, but that the same biological processes are involved. Moreover, we found that histotype-specificity seems to be principally guided by small non-coding RNAs as most of them modulate the cross-talk between different areas of the network with a histotype-specific expression.

Secondly, our results indicate a central role of cAMP-PKA-CREB1 signaling axis as the main pathway associated with histotype specificity. This pathway promotes the expression of cAMP-dependent target genes and requires the activation of CREB1 by cAMP-dependent protein kinase A (PKA). This signal has been already linked to the platinum resistance in advanced ovarian cancer by Dimitrova et al. [24] showing that patients with high cAMP have low progression-free survival.

cAMP-PKA-CREB1 signaling is linked to a large range of other biological signals such as metabolic pathways, BAD oncogene, and, exclusively in Cc, the regulation of intracellular availability of Calcium. It is clear from the network topology that miRNAs elements play central role in histotype-specificities modulating the interplay between different areas of the network such as RAC1, MAPK signaling pathway, cell cycle, and growing factor receptors.

We confirm the role of Cc miRNA biomarkers miR-30a-3p and miR-30a-5p [16] in cell cycle regulation, as well as of Muc miRNA biomarkers, miR-192 and miR-194 [17], in regulating multiple steps of cell cycle, Activin and Inhibins pathways, and Integrins.

Apart from their role in physiological events, integrins are also involved in many pathological conditions such as inflammation and tumor progression. Here, different histotypes are characterized by the deregulations of specific integrins: ITGA1, ITGA11, ITGB1 are downregulated in Ser samples while ITGA10, ITGB4 in Cc, ITGB8 in Muc and ITGB3 in End. The involvement of ITGA1 at least in serous histotype suggests an MYC dependent regulation. Very recently, a new competing enhancer regulatory mechanism between PVT1 and MYC has been demonstrated [25]. PVT1 promoter partially insulates downstream enhancer elements from regulating MYC expression, and thus functions as an intra-TAD boundary element. In this way, PVT1 expression that we found associated with poor prognosis [15], impairs MYC expression that at the same time is a key regulator of ITGA1 [26] downregulated in high and low grade Ser samples. ITGA1 is found to be a key player of cell adhesion-mediated drug resistance in ovarian cancer [27], thus its strong downregulation in Ser histotype suggests a more therapy-sensitive phenotype of serous ovarian tumors compared to other histotypes.

The only link to the immune system is reported through the presence of SOCS2 that is a target of miR-192 and is downregulated in Ser samples. SOCS2 is a negative regulator of the IFN-gamma signaling pathway, which, in turn, is a key regulator of balance between immune activation and immune suppression [28]. Specifically, loss of SOCS2 enables robust tumor-immune rejection by expanding DC-based T cell priming and antigen-specific adaptive immunity. In agreement with our previous observations, our network suggests a higher immune-reactive behavior of Ser (with low levels of SOCS2 for both high and low grade serous) and Muc (with high levels of miR-194-5p that targets SOCS2) than in other histotypes.

### 2.5. Histotype-Specific Transcripts Validation

To validate our network results, a list of 53 (30 miRNAs and 23 genes) transcripts were selected and their expression values were assessed using qRT-PCR in the training set. The selection was made according to literature evidence, expression differences across histotypes, and topological position within the network.

In the training set, a total of 20 molecular elements (38%) were confirmed as histotype-specific (7 over 23 genes and 13 over 30 miRNAs) (Supplementary Material 5). Apart from the already published mucinous (miR-192 and miR-194) and clear cell (miR-30a-3p and miR-30a-5p) miRNA biomarkers [16], we confirm some known coding and non coding elements known to be involved in tumor progression e.g., MDM2, ESR1, E2F3, Cyclin Dependent Kinases (CDKN1A, CDKN2A, CDK4 and CDK6), hsa-miR-214-3p and hsa-miR-29a/b/c, and hsa-let-7a.

These 20 elements were further tested in an independent validation set (Table 2 and Supplementary Material 5). Ten out of the 13 miRNAs (77%) and 5 of the 8 genes (63%) were validated as histotype specific (Figure 3B and Supplementary Material 5). As novel biomarkers, we found miR-214-3p and let7a-5p showing significantly high levels in Muc and SerLow histotypes, miR-96 showing low levels in Muc and SerLow, and mir-29b that is highly expressed only in SerLow subtypes. On the other hand, looking at the coding genes, we found that Muc histotype has a significantly lower and higher expression levels of respectively MDM2 and CDKN2A with respect to other subtypes, CDK6 is lowly expressed in Muc and low grade subtypes, while CDKN1A is upregulated in low grade serous samples. Instead, the Cc subtype is characterized by low levels of E2F3.

## 3. Materials and Methods

### 3.1. Patient Cohorts

Primary tumor tissue of patients with a diagnosis of stage I EOC was included in the study. Analysis protocols and sample collection procedures were carried out in accordance with the recommendations of local scientific ethical committees. A written informed consent was obtained from all the patients enrolled in the study, which has been conducted following the Declaration of Helsinki set of principles.

At the time of surgery, tumor tissue samples were sharp dissected and snap frozen in liquid nitrogen within 15 min from restriction, and then stored at −80 °C.

Training set was composed of 76 frozen biopsies conserved in the tissue bank collected since September 1992 and located at the Department of Oncology, IRCCS Mario Negri Institute, Milano, Italy (study number: 1065 date of Ethical commitee approval 10 November 2015). Biopsies were collected from patients who underwent surgery for EOC at the Obstetrics and Gynaecology Department, San Gerardo Hospital, Monza, Italy, as described by Marchini et al. (Marchini, Lancet Oncol. 2011).

A validation set was composed of 132 frozen samples: (i) 26 tumors’ tissues from the tissue bank collected since March 2003 at the A. Nocivelli Institute for Molecular Medicine, Division of Gynecologic Oncology, University of Brescia, Italy. Samples derived from patients who underwent primary surgery for EOC at the Obstetrics and Gynecology Department, Spedali Civili, Brescia, Italy (study reference number: 548, date Ethical Commitee approval 5 April 2011); (ii) 47 tissue sample belonging to a tissues collection that were collected since January 1992 and available at the Department of Gynaecology-Oncology, University of Torino, Torino, Italy (ii) and finally 59 additional samples from the tissue bank at the Department of Oncology, IRCCS Mario Negri Institute, Milano, Italy.

Histopathological and clinical characteristics of the 208 enrolled patients are summarized in Table 1.

### 3.2. Expression Analyses

Frozen tissues specimens (30 mg) were homogenized in an TissueLyser LT (Qiagen, Milan, Italy) and total ribonucleicacid (RNA) purified using RNeasy Mini Kit isolation system (Qiagen), following manufacturers’ protocols. Total RNA concentration and proteins contamination were determined by a Nanodrop spectrophotometer (Nanodrop Technologies, Ambion, Waltham, MA, USA). Only samples with a RNA integrity number (RIN) larger than six and a Nanodrop A260:280 ratio between 1.8 and 2.1 were further processed and aliquots stored at −80 °C until use. Array experiments were performed using standard procedures as previously published by Calura et al. [16]. Briefly, 100 ng of total RNA was reverse transcribed into Cy3-labeled cRNA using LowInput QuickAmp labeling kit (Agilent Technologies, Palo Alto, CA, USA) and hybridized with a RNA labeling and hybridization kit according to the manufacturer’s instructions (Agilent Technologies). miRNA extraction, labeling and hybridization using commercially available G4470B human miRNA Microarray kit (Agilent) were performed as previously described [16], raw data have been submitted to ArrayExpress (E-MTAB-1067). For gene expression measurements, we used the commercially available G4851B human whole GE Microarray kit (SurePrint G3 Human Gene Expression 8×60K v2 Microarray Kit Agilent Technologies) which consists of 60 K features printed in an 8-plex format. The arrays were washed and scanned with a laser confocal scanner (G2565B, Agilent Technologies) according to the manufacturer’s instructions. mRNA microarrays underwent standard post-hybridization processing and the intensities of fluorescence were calculated by Feature Extraction software v11 (Agilent Technologies). Gene expression raw data have been submitted to ArrayExpress (E-MTAB-1814). Raw data (mRNA and miRNA) were pre-processed to filter out those probes with more than 40% of measurements below the signal-to-noise threshold. Filtered data were normalized using quantile normalization [29].

### 3.3. Ovarian Cancer Molecular Signature Identification

The samples were classified according to a number of previously-published signatures in ovarian cancer as described by Helland et al. [30], Bentink et al. [31], Verhaak et al. [32], and Konecny et al. [33] representing four different molecular subtypes as previously described by Tothill and colleagues [18]. These molecular classifications were grouped in a single consensus classifier of 71 genes which was then trained on the *MetaGxOvarian* data set using a random forest approach. The trained classifier was used on the samples, selecting for each the molecular signature with the highest probability. The computation was performed with the *consensusOV* R package available on *Bioconductor*.

### 3.4. Immuno-Phenotype Analyses

The absolute quantification of immune system cell fractions from bulk tissue gene expression profiles has been provided using CIBERSORT [34]. Normalized gene expression matrix has been submitted to a CIBERSORT web tool using an LM22 signature file, which can define 22 subtypes of immune cells with the preset signature matrix at 1000 permutations.

The immunophenograms and immunophenoscores were constructed as proposed by Charoentong et al. using the R code distributed by authors [9]. The immunophenogram is a graphical representation of the patient specific immunophenotype inferred using the expression of a panel of genes representative of specific immune cell types. This graphical representation was adopted by [9] to describe immune cell types activation (red) and inactivation (blu) at the sample level. The wheel represents a sample, and it is divided into sections. The external sections represent the list of the 26 determinants (20 single factors, MHC molecules, immunoinhibitors, and immunostimulators and six cell types, activated CD4+ T cells, activated CD8+ T cells, effector memory CD4+ T cells and effector memory CD8+ T cells, Tregs, and MDSCs) identified by [9] as the best consensus features discriminating between patients with high and low cytolytic activity. These 26 determinants are grouped according to their functional properties into 4 classes (the four internal sections of the wheel): effector cells (activated CD4+ T cells, activated CD8+ T cells, effector memory CD4+ T cells, and effector memory CD8+ T cells), suppressive cells (Tregs and MDSCs), MHC-related molecules, and checkpoints or immunomodulators. The color of the sections is proportional to their aggregated expression Z-score (red for the active and blue for the inactive ones). The scaled aggregated Z-score of all the determinants generate the patient IPS.

*t*-test was used to assess differences among the z-score of the four sets of determinants. A Kruskal–Wallis rank sum test was used to assess differences of IPS across histotypes. We used a pheatmap CRAN R package to provide cluster analyses [35]. We used a ggplot2 R package for all the box-plots and histograms [36]. Kaplan–Meier curves of IPS patient groups have been provided using a survival R package.

### 3.5. Network Analysis

Gene and microRNA integrated topological pathway analysis has been performed using Micrographite pipeline [17] and Clipper [12]. KEGG pathways was used through Graphite R package [37,38]. Two different batches of miRNA target interactions are considered: the in silico predicted interactions and the validated ones. The validated miRNA target interactions are derived from mirTarBase [39], miRecords [40], and a manual bibliographic research, selecting only the interactions validated by a reporter assay. Specifically, in the ovarian cancer dataset, we used TargetScan predictions (with *Pct* ≥ 0.8) and predicted miRNA-mRNA couples with a Pearson correlation coefficient |*r*| ≥ 0.4 and *q*-value ≤ 0.05. Four pathway tests have been performed through pairwise comparison between each histotype and all the others, then resulting networks were merged together. The network proxy of survival identified in Calura et al. [13] was also added due to the large overlap with the histotype analysis.

### 3.6. qRT-PCR Data Analysis

Gene and miRNAs expression levels were validated by qRT-PCR on both training (array set) and validation sets of patients. qRT-PCR have been performed using Sybr Green protocol (Qiagen, Milano, Italy) on an Applied Biosystems 7900HT instrument (Waltham, MA, USA). Experiments were run in triplicate, using 384-well reaction plates in an automatic liquid handling station (epMotion 5075LH; Eppendorf, Milano, Italy). Raw data was generated with Sodium dodecylsulphate (SDS) Relative Quantification software (version 2.3; Ambion-ABI), data were normalized using the geometric mean of the four independent housekeeping controls (for miRNAs: RNU6B, SNORD61, SNORD72, SNORD68; for genes: ACTB, B2M, PPIA and HPRT1). Two-sided Student’s *t*-test were used to verify between groups’ mean differences, then the *p*-value underwent Benjamini–Hochberg FDR-controlling procedure, and only comparison with *adjusted* *p*-value ≤ 0.1 was considered statistically significant and reported in Table 2.

## 4. Conclusions

In this work, using in silico analyses, we describe the molecular signatures and biological processes engaged in stage I EOC, with the aim to give a global view of the tumor heterogeneity with a focus on histotype specificity.

Using gene expression data on a sizeble stage I EOC multicentric cohort, we found that a SerHigh subtype shows more aggressive phenotypes with respect to the other subtypes as many genes involved in cell proliferation, migration, and DNA repair are transcriptionally altered. Moreover, our results indicate that single genes rather than pathways are responsible for histotypes’ specificities and that miRNAs seem to be key modulators of these common pathways. The cAMP-PKA-CREB1 signaling axis plays a central role in histotype differentiation together with metabolic pathway, cell cycle regulation MAPK signaling, and apoptosis.

Interestingly, immune response seems to be, at least in part, involved in histotype differences, as a high immune-reactive behavior in low grade serous and mucinous samples was observed compared to other histotypes. In addition, we found differences between low and high grade serous samples as the latter seems to be characterized by a lower amount of infiltrated immune cells with a less immune-reactive phenotype.

Similar findings but using a standardised haematoxylin and eosin staining approach on advanced EOC samples were obtained by [41]. The authors showed that stromal and intratumoral tumor-infiltrating lymphocyte levels correlated with tumor histological subtype as higher levels of immune infiltration were observed in serous ovarian tumors than in other histotypes. Moreover, they showed that increased levels of both intratumoral and stromal tumor-infiltrating lymphocytes were associated with a better prognosis.

In general, we found high levels of major histocompatibility complex (MHC) genes in all samples confirming that these tumors are characterized by an intact antigen processing apparatus and by abundant cytotoxic T lymphocyte (CTL) infiltration. These features, however, do not appear to translate in beneficial effects on patients survival as already reported by Gooden and collegues (2011).

Taken together, our results suggest that the immune system, in combination with other signaling cascades, is one of the molecular players in early stage ovarian cancer supporting the idea that different subtypes are variably immunogenic.

If our results might contribute to define molecular specificities and similarities of stage I EOC subtypes, they certainly need further validations (i) increasing the sample size especially for low grade serous subtype that in our cohort is poorly represented and (ii) with additional immune-specific assays to confirm cell type composition. Moreover it is clear that, although computational methods for the study of tumor microenvironment from bulk data are becoming increasingly efficient, the interpretation of their results is still challenging. In this context, the emerging technologies for single-cell and spatially resolved transcriptomics are boosting cancer genomic research towards new perspectives allowing a better understanding of the cellular composition and structure of tumors and how these contribute to the molecular subtype specificities.

## Figures and Tables

**Table 1:** Main clinical and histopathological characteristics of patients divided into training and validation sets (the percentage values are in brackets).

Clinical Annotation	Training Set	Validation Set
**HISTOTYPES**
Clear cell	16 (21%)	22 (16.6%)
Endometroid	19 (25%)	55 (41.7%)
Mucinous	17 (22.4%)	21 (15.9%)
Serous high-grade	16 (21.0%)	26 (76.4%)
Serous low-grade	8 (10.6%)	8 (23.5%)
**GRADES**
G1	18 (23.7%)	32 (32.6%)
G2	18 (23.7%)	31 (31.7%)
G3	38 (50.0%)	35 (35.7%)
NA	2 (2.6%)	0 (0%)
**RELAPSING**
No	55 (72.4%)	105 (79.5%)
Yes	21 (27.6%)	24 (18.2%)
NA	0 (0%)	3 (2.3%)
**VITAL STATUS AT THE LAST FOLLOW UP**
Alive	57 (75%)	102 (77.3%)
Dead of EOC	15 (19.8%)	9 (6.8%)
Dead of other cause	2 (2.6%)	13 (9.9%)
Unknown	0 (0%)	4 (3%)
Awarded	2 (2.6%)	4 (3%)
**FIGO SUBSTAGE**
A	25 (32.9%)	27 (20.5%)
B	4 (5.3%)	6 (4.5%)
C	47 (61.8%)	52 (39.4%)
NA	0 (0%)	47 (35.6%)
**Mean age in years [min-max]**	53 [21–82]	55 [17–80]
**Mean follow up in years [min-max]**	9 [1–18]	6 [0–17]
**Total number of patients**	76	132

**Table 2:** *t*-test *p*-value and adjusted *p*-value of qRT-PCR expression values in validation set samples.

	Validation Set																			
	Muc vs SerHigh		Muc vs SerLow		Muc vs End		Muc vs Cc		SerHigh vs End		SerLow vs End		SerHigh vs Cc		SerLow vs Cc		End vs Cc		SerH vs SerL	
	p	adj-p	p	adj-p	p	adj-p	p	adj-p	p	adj-p	p	adj-p	p	adj-p	p	adj-p	p	adj-p	p	adj-p
hsa-let-7a-5p	0.0012	0.0091	0.9849	0.9863	0.0602	0.3635	0.0016	0.0074	0.0520	0.2758	0.1683	0.5946	0.4018	0.6980	0.0169	0.0805	0.1688	0.5215	0.0221	0.2328
hsa-miR-192-5p	0.0000	0.0000	0.0000	0.0000	0.0000	0.0000	0.0000	0.0000	0.6842	0.9218	0.8806	0.9527	0.1767	0.6247	0.7796	0.8982	0.6812	0.8535	0.3771	0.7724
hsa-miR-194-5p	0.0000	0.0000	0.0000	0.0000	0.0000	0.0000	0.0000	0.0000	0.8083	0.9218	0.7085	0.8940	0.1768	0.6247	0.9347	0.9527	0.5082	0.7506	0.4415	0.7784
hsa-miR-214-3p	0.0000	0.0005	0.6570	0.9118	0.0014	0.0153	0.0000	0.0001	0.3073	0.8143	0.0040	0.1054	0.8778	0.9481	0.0000	0.0001	0.2122	0.5367	0.0002	0.0096
hsa-miR-26a-5p	0.0072	0.0346	0.9291	0.9863	0.0790	0.4037	0.0033	0.0135	0.3287	0.8280	0.1551	0.5874	0.9999	0.9999	0.0095	0.0630	0.2958	0.6990	0.0283	0.2328
hsa-miR-29a-3p	0.1899	0.4575	0.0512	0.3874	0.3262	0.6668	0.0957	0.2205	0.7376	0.9218	0.0132	0.1754	0.7564	0.9481	0.0025	0.0187	0.5136	0.7506	0.0070	0.1189
hsa-miR-29b-3p	0.0044	0.0260	0.1692	0.9118	0.0031	0.0274	0.0003	0.0016	0.9426	0.9796	0.0024	0.1054	0.4083	0.6980	0.0007	0.0097	0.4482	0.7506	0.0035	0.0917
hsa-miR-30a-3p	0.8097	0.9331	0.5548	0.9118	0.8213	0.9572	0.0000	0.0000	0.9633	0.9818	0.7056	0.8940	0.0000	0.0000	0.0000	0.0000	0.0000	0.0000	0.4771	0.7784
hsa-miR-30a-5p	0.9686	0.9881	0.5980	0.9118	0.7880	0.9492	0.0000	0.0000	0.7934	0.9218	0.6702	0.8940	0.0000	0.0000	0.0000	0.0000	0.0000	0.0000	0.5494	0.8089
hsa-miR-96-5p	0.4640	0.6767	0.9863	0.9863	0.5985	0.8572	0.0003	0.0018	0.7916	0.9218	0.6623	0.8940	0.0011	0.0140	0.0020	0.0176	0.0004	0.0058	0.5140	0.7784
CDK6	0.0004	0.0032	0.7787	0.9863	0.0124	0.0940	0.0017	0.0074	0.4418	0.9218	0.0986	0.4751	0.9367	0.9547	0.0284	0.1157	0.5240	0.7506	0.0090	0.1189
CDKN1A	0.0064	0.0339	0.5615	0.9118	0.4863	0.8054	0.0217	0.0715	0.0093	0.1910	0.8607	0.9527	0.2890	0.6831	0.2496	0.4267	0.0413	0.2739	0.1272	0.4496
CDKN2A	0.0000	0.0000	0.0000	0.0000	0.0000	0.0000	0.0000	0.0000	0.2831	0.7896	0.7871	0.9426	0.0129	0.0857	0.0463	0.1504	0.0020	0.0209	0.5659	0.8107
E2F3	0.3567	0.6301	0.8805	0.9863	0.3187	0.6668	0.0001	0.0004	0.0788	0.3797	0.4780	0.8940	0.0028	0.0249	0.0118	0.0697	0.0000	0.0003	0.6869	0.8307
MDM2	0.0000	0.0000	0.0001	0.0016	0.0000	0.0000	0.0000	0.0000	0.5109	0.9218	0.5543	0.8940	0.0008	0.0140	0.1640	0.3858	0.0138	0.1218	0.2344	0.5730

## Supplementary Materials

Supplementary materials are available online at https://www.mdpi.com/2073-4409/8/12/1554/s1.

## Abbreviations

The following abbreviations are used in this manuscript:

EOC	Epithelial Ovarian Cancer
FIGO	International Federation of Gynecological and Obstetrics
IPS	Immunophenoscore
IPG	Immunophenogram
KM	Kaplan–Meier
MUC	Mucinous
SER HIGH	High Grade Serous
SER LOW	Low Grade Serous
CC	Clear Cell
END	Endometrioid
DIF	Differentiated subtype
MES	Mesenchymal subtype
MHC	Major Histocompatibility Complex
CTL	Cytotoxic T Lymphocyte
HLA	Human Leukocyte Antigen
GO	Gene Ontology
IMR	Immunoreactive subtype
PRO	Proliferative subtype
