# Supplementary material for: Transcriptional Characterization of Stage I Epithelial Ovarian Cancer: A Multicentric Study
Source: Cells. 2019 Dec 1;8(12):1554. doi: 10.3390/cells8121554 (PMC6952972; doi:10.3390/cells8121554)
Supplement: Supplementary file 1 [file cells-08-01554-s001.zip › SuppMat1.pdf]

Supplementary File 1: Stage I classification using the TCGA expression subtypes

Contents

Classification based on Tothill's signatures 1

Association between signatures and histotypes 3

Association between signature and tumor grade 4

Association between signature and FIGO 5

Association between signature and relapse 6

Classification based on Tothill's signatures

Table 1: Consensus of expression signature

	IMR_consensus	DIF_consensus	PRO_consensus	MES_consensus	calculated.subtype
P562	0.588	0.290	0.078	0.044	IMR_consensus
P353	0.016	0.974	0.004	0.006	DIF_consensus
P957	0.958	0.020	0.002	0.020	IMR_consensus
P111	0.218	0.256	0.452	0.074	PRO_consensus
P323	0.326	0.182	0.182	0.310	IMR_consensus
P509	0.142	0.084	0.148	0.626	MES_consensus
P884	0.916	0.064	0.002	0.018	IMR_consensus
P928	0.046	0.852	0.086	0.016	DIF_consensus
P302	0.762	0.130	0.024	0.084	IMR_consensus
P356	0.146	0.602	0.188	0.064	DIF_consensus
P949	0.596	0.374	0.014	0.016	IMR_consensus
P940	0.026	0.958	0.008	0.008	DIF_consensus
P832	0.016	0.000	0.004	0.980	MES_consensus
P203	0.228	0.316	0.182	0.274	DIF_consensus
P416	0.298	0.186	0.090	0.426	MES_consensus
P556	0.002	0.000	0.008	0.990	MES_consensus
P969	0.034	0.960	0.004	0.002	DIF_consensus
P490	0.070	0.876	0.030	0.024	DIF_consensus
P344	0.294	0.556	0.082	0.068	DIF_consensus
P321	0.908	0.042	0.002	0.048	IMR_consensus
P816	0.102	0.710	0.080	0.108	DIF_consensus
P765	0.380	0.572	0.024	0.024	DIF_consensus

P426	0.344	0.078	0.010	0.568	MES_consensus
P116	0.158	0.126	0.366	0.350	PRO_consensus
P531	0.204	0.324	0.222	0.250	DIF_consensus
P615	0.130	0.386	0.348	0.136	DIF_consensus
P811	0.090	0.522	0.232	0.156	DIF_consensus
P517	0.102	0.780	0.054	0.064	DIF_consensus
P283	0.386	0.068	0.018	0.528	MES_consensus
P314	0.096	0.072	0.128	0.704	MES_consensus
P192	0.922	0.046	0.002	0.030	IMR_consensus
P397	0.344	0.418	0.162	0.076	DIF_consensus
P115	0.462	0.036	0.026	0.476	MES_consensus
P142	0.732	0.184	0.060	0.024	IMR_consensus
P973	0.996	0.002	0.000	0.002	IMR_consensus
P140	0.006	0.974	0.018	0.002	DIF_consensus
P526	0.174	0.626	0.094	0.106	DIF_consensus
P794	0.166	0.150	0.110	0.574	MES_consensus
P131	0.028	0.024	0.890	0.058	PRO_consensus
P239	0.148	0.238	0.240	0.374	MES_consensus
P547	0.834	0.062	0.006	0.098	IMR_consensus
P240	0.066	0.052	0.256	0.626	MES_consensus
P127	0.754	0.220	0.016	0.010	IMR_consensus
P463	0.116	0.032	0.064	0.788	MES_consensus
P569	0.116	0.074	0.444	0.366	PRO_consensus
P790	0.574	0.344	0.030	0.052	IMR_consensus
P315	0.140	0.556	0.236	0.068	DIF_consensus
P797	0.298	0.154	0.176	0.372	MES_consensus
P958	0.266	0.400	0.138	0.196	DIF_consensus
P872	0.076	0.148	0.224	0.552	MES_consensus
P716	0.066	0.468	0.256	0.210	DIF_consensus
P202	0.448	0.242	0.100	0.210	IMR_consensus
P310	0.716	0.214	0.028	0.042	IMR_consensus
P304	0.146	0.350	0.124	0.380	MES_consensus
P936	0.118	0.180	0.594	0.108	PRO_consensus
P782	0.086	0.836	0.046	0.032	DIF_consensus
P721	0.428	0.234	0.108	0.230	IMR_consensus
P398	0.032	0.002	0.000	0.966	MES_consensus
P941	0.118	0.450	0.270	0.162	DIF_consensus
P442	0.822	0.090	0.024	0.064	IMR_consensus
P291	0.368	0.320	0.204	0.108	IMR_consensus
P592	0.590	0.210	0.070	0.130	IMR_consensus
P396	0.236	0.280	0.216	0.268	DIF_consensus
P130	0.072	0.082	0.494	0.352	PRO_consensus
P189	0.030	0.016	0.156	0.798	MES_consensus
P459	0.612	0.248	0.094	0.046	IMR_consensus

P642	0.594	0.212	0.136	0.058	IMR_consensus
P860	0.038	0.070	0.876	0.016	PRO_consensus
P599	0.028	0.058	0.880	0.034	PRO_consensus
P287	0.060	0.090	0.118	0.732	MES_consensus
P791	0.132	0.518	0.248	0.102	DIF_consensus
P831	0.082	0.008	0.014	0.896	MES_consensus
P211	0.036	0.914	0.034	0.016	DIF_consensus
P849	0.084	0.128	0.652	0.136	PRO_consensus
P349	0.136	0.736	0.066	0.062	DIF_consensus
P745	0.070	0.186	0.576	0.168	PRO_consensus

Association between signatures and histotypes

Table 2: Contingency table for histotypes.

	DIF_consensus	IMR_consensus	MES_consensus	PRO_consensus
Cc	3	6	6	1
End	6	2	5	6
Muc	6	3	7	1
SerHigh	4	9	1	2
SerLow	6	1	0	0

Chi-square p-value 0.0037847.

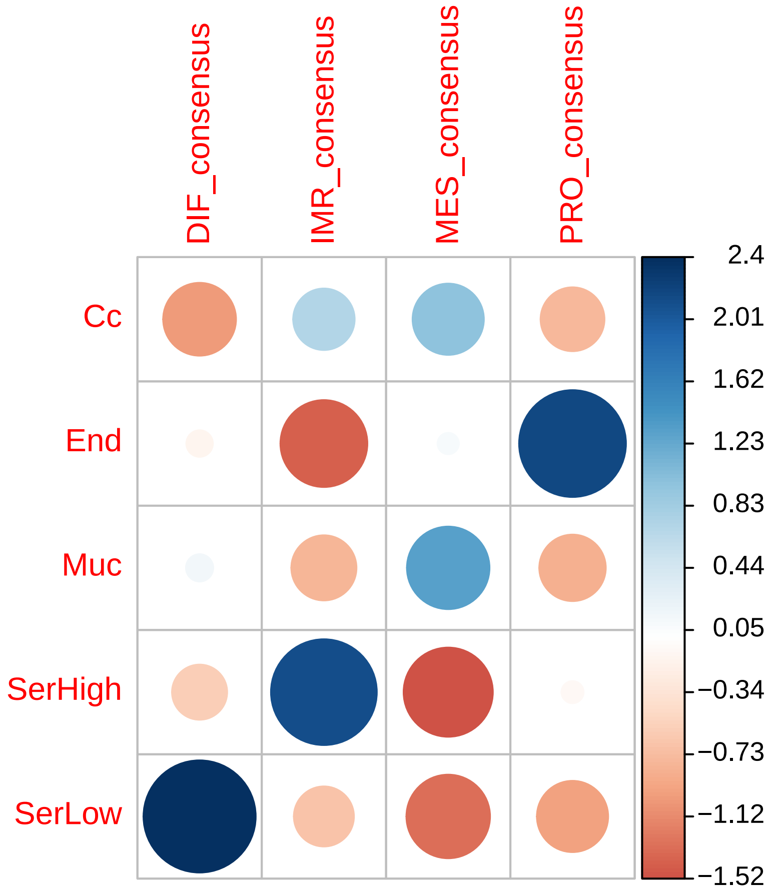

Figure 1: Graphical representation of Chi-square residuals. For a given cell, the size of the circle is proportional to the amount of the cell contribution. Blu positive associations, red negative associations

Association between signature and tumor grade

Table 3: Contingency table for tumor grade.

	DIF_consensus	IMR_consensus	MES_consensus	PRO_consensus
G1	1	1	7	1
G2	9	3	2	4
G3	3	7	9	3
High	4	9	1	2
Low	6	1	0	0

Chi-square p-value 0.0003.

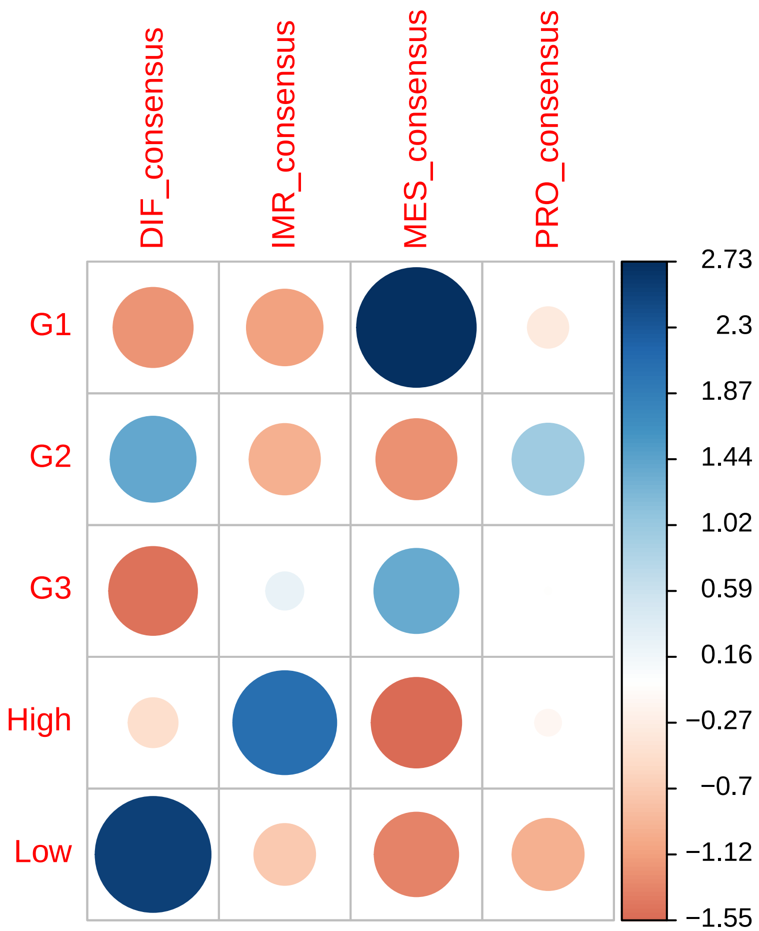

Figure 2: Graphical representation of Chi-square residuals. For a given cell, the size of the circle is proportional to the amount of the cell contribution. Blu positive associations, red negative associations

Association between signature and FIGO

Table 4: Contingency table for FIGO classification.

	DIF_consensus	IMR_consensus	MES_consensus	PRO_consensus
a	10	5	9	1
b	1	2	1	0
c	15	14	9	9

Chi-square p-value 0.3206333.

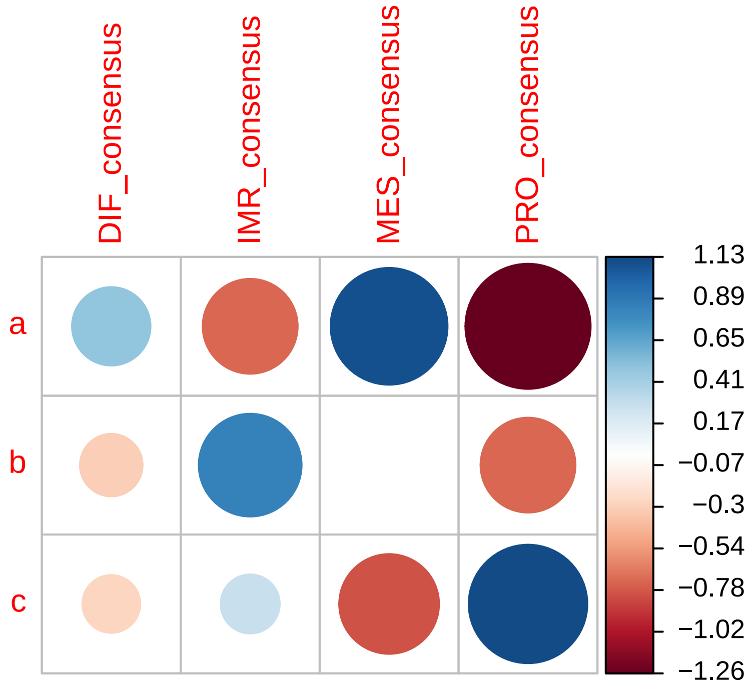

Figure 3: Graphical representation of Chi-square residuals. For a given cell, the size of the circle is proportional to the amount of the cell contribution. Blu positive associations, red negative associations

Association between signature and relapse

Table 5: Contingency table for relapse events.

	DIF_consensus	IMR_consensus	MES_consensus	PRO_consensus
N	22	14	15	4
Y	4	7	4	6

Chi-square p-value 0.0472187.

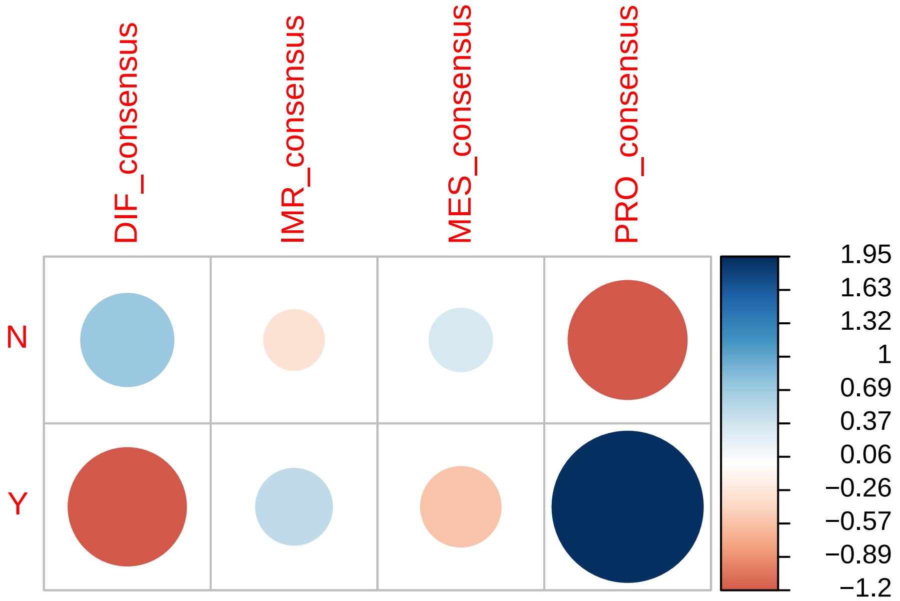

Figure 4: Graphical representation of Chi-square residuals. For a given cell, the size of the circle is proportional to the amount of the cell contribution. Blu positive associations, red negative associations
