# Supplementary material for: Transcriptional Characterization of Stage I Epithelial Ovarian Cancer: A Multicentric Study
Source: Cells. 2019 Dec 1;8(12):1554. doi: 10.3390/cells8121554 (PMC6952972; doi:10.3390/cells8121554)
Supplement: Supplementary file 1 [file cells-08-01554-s001.zip › SuppMat2.pdf]

Supplementary Material 2: quantification of immune system cell fractions from bulk tissue gene expression

Contents

Heatmaps of cybersort 2
Immune cell aboundancies across histopathological annotations 2
Plot frequencies of cell proportion 2
Anova tests 3
Summary of significant Anova comparisons across histo-pathological annotations of immune cell deconvolution and plots 4
Survival associations 6
Anova test with the total amount of immune cells for patient 8
Survival association with the total amount of immune cells for patient 9

Heatmaps of cybersort

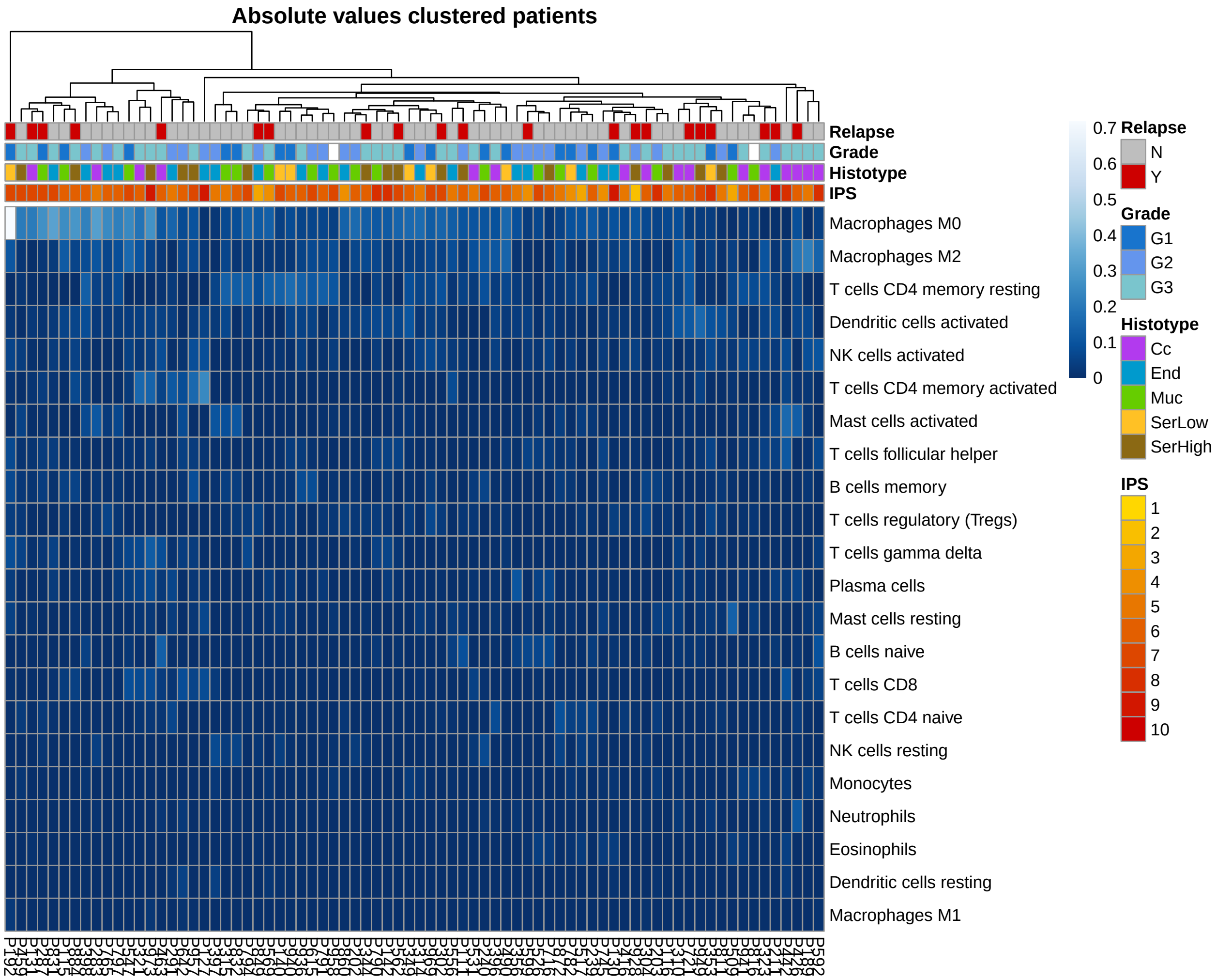

Figure 1: Cybersort heatmap and clustering of patients

Immune cell aboundancies across histopathological annotations

Plot frequencies of cell proportion

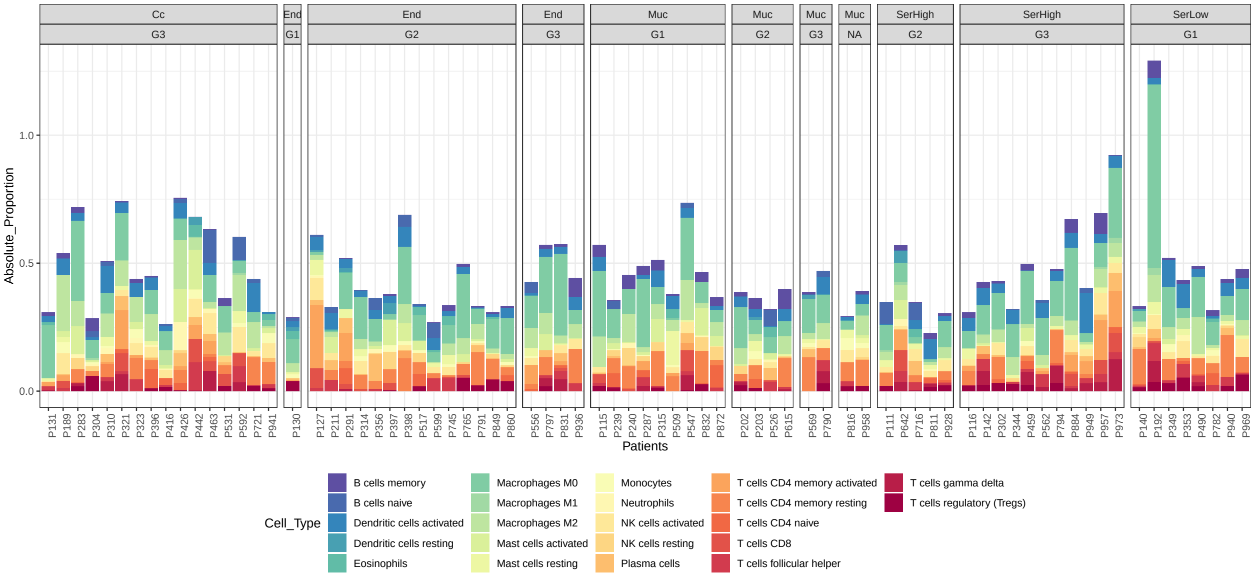

Anova tests

Anova comparisons Table1 across histo-pathological annotations of immune cell deconvolution absolute aboundancies (p_anova). Tukey multiple comparisons of means 95% family-wise confidence level: "diff" gives the difference in the observed means; "lwr" gives the lower end point of the interval; "upr" gives the upper end point and "p_adj" gives the p-value after adjustment for the multiple comparisons. A summary is reported in Table2

Table 1: Anova comparisons across histo-pathological annotations of immune cell deconvolution absolute aboundancies.

cellType	clin	comp	diff	lwr	upr	p_adj	p_anova
T cells regulatory (Tregs)	Histotype	End-Cc	0.00200	-0.01292	0.01692	0.99572	0.0455
T cells regulatory (Tregs)	Histotype	Muc-Cc	0.00043	-0.01488	0.01575	0.99999	0.0455
T cells regulatory (Tregs)	Histotype	SerHigh-Cc	0.00396	-0.01159	0.01950	0.95299	0.0455
T cells regulatory (Tregs)	Histotype	SerLow-Cc	0.01981	0.00077	0.03885	0.03732	0.0455
T cells regulatory (Tregs)	Histotype	Muc-End	-0.00156	-0.01624	0.01312	0.99824	0.0455
T cells regulatory (Tregs)	Histotype	SerHigh-End	0.00196	-0.01296	0.01688	0.99601	0.0455
T cells regulatory (Tregs)	Histotype	SerLow-End	0.01782	-0.00072	0.03635	0.06532	0.0455
T cells regulatory (Tregs)	Histotype	SerHigh-Muc	0.00352	-0.01179	0.01884	0.96720	0.0455
T cells regulatory (Tregs)	Histotype	SerLow-Muc	0.01938	0.00053	0.03823	0.04091	0.0455
T cells regulatory (Tregs)	Histotype	SerLow-SerHigh	0.01586	-0.00318	0.03489	0.14721	0.0455
NK cells resting	Histotype	SerHigh-Muc	-0.01632	-0.03174	-0.00089	0.03289	0.0627
Macrophages M2	Histotype	End-Cc	-0.05072	-0.09077	-0.01066	0.00612	0.0065
Macrophages M2	Histotype	Muc-Cc	-0.03172	-0.07283	0.00940	0.20734	0.0065
Macrophages M2	Histotype	SerHigh-Cc	-0.04909	-0.09083	-0.00736	0.01304	0.0065
Macrophages M2	Histotype	SerLow-Cc	-0.02969	-0.08081	0.02142	0.48612	0.0065
Macrophages M2	Histotype	Muc-End	0.01900	-0.02041	0.05841	0.66143	0.0065
Macrophages M2	Histotype	SerHigh-End	0.00163	-0.03843	0.04168	0.99996	0.0065
Macrophages M2	Histotype	SerLow-End	0.02103	-0.02873	0.07078	0.76106	0.0065
Macrophages M2	Histotype	SerHigh-Muc	-0.01738	-0.05849	0.02374	0.76110	0.0065
Macrophages M2	Histotype	SerLow-Muc	0.00202	-0.04859	0.05264	0.99996	0.0065
Macrophages M2	Histotype	SerLow-SerHigh	0.01940	-0.03172	0.07052	0.82502	0.0065

Table 1: Anova comparisons across histo-pathological annotations of immune cell deconvolution absolute aboundancies. *(continued)*

cellType	clin	comp	diff	lwr	upr	p_adj	p_anova
T cells gamma delta	Grade	G2-G1	-0.00653	-0.02437	0.01131	0.65688	0.0129
T cells gamma delta	Grade	G3-G1	0.01246	-0.00415	0.02907	0.17820	0.0129
T cells gamma delta	Grade	G3-G2	0.01899	0.00360	0.03439	0.01173	0.0129
NK cells resting	Grade	G2-G1	-0.00868	-0.02070	0.00334	0.20189	0.0259
NK cells resting	Grade	G3-G1	-0.01297	-0.02416	-0.00177	0.01920	0.0259
NK cells resting	Grade	G3-G2	-0.00429	-0.01467	0.00609	0.58621	0.0259
Macrophages M2	Grade	G2-G1	-0.03039	-0.06292	0.00215	0.07207	0.0105
Macrophages M2	Grade	G3-G1	0.00489	-0.02541	0.03518	0.92121	0.0105
Macrophages M2	Grade	G3-G2	0.03527	0.00719	0.06336	0.01008	0.0105

Summary of significant Anova comparisons across histo-pathological annotations of immune cell deconvolution and plots

Table 2: Summary of significant results.

cellType	clin	p_anova
T cells regulatory (Tregs)	Histotype	0.0455
NK cells resting	Histotype	0.0627
Macrophages M2	Histotype	0.0065
T cells gamma delta	Grade	0.0129
NK cells resting	Grade	0.0259
Macrophages M2	Grade	0.0105

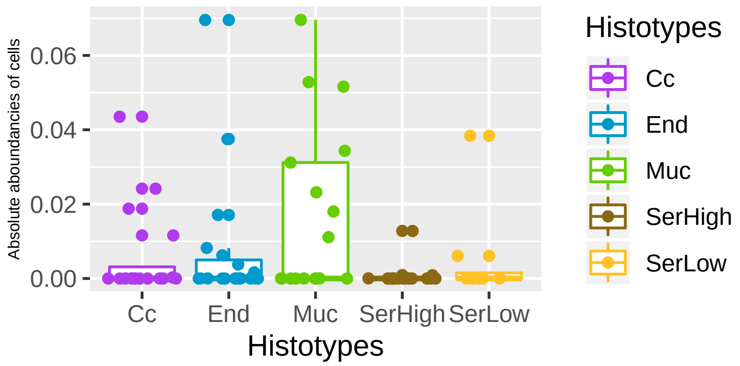

Figure 2: NK Cells Resting across Histotype

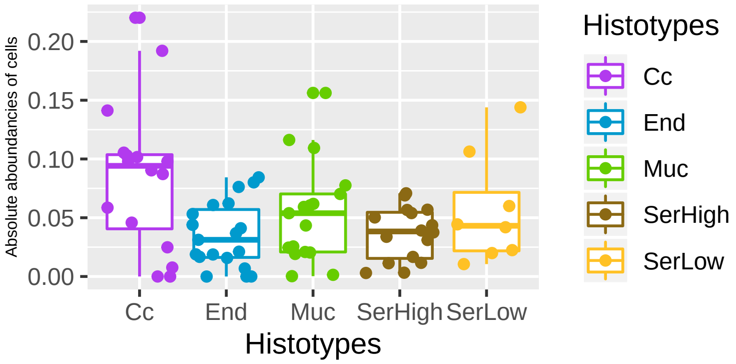

Figure 3: Macrophages M2 across Histotype

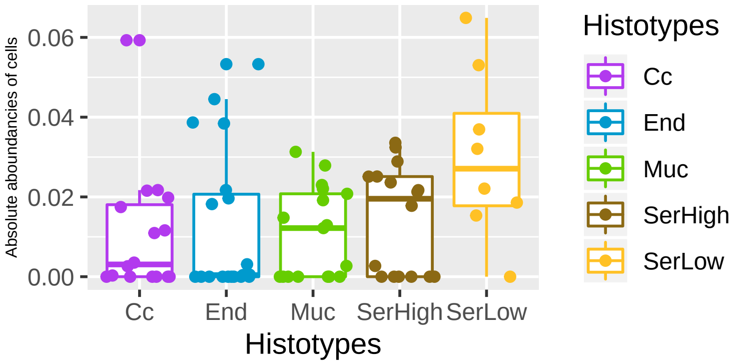

Figure 4: T cells regulatory (Tregs) across Histotype

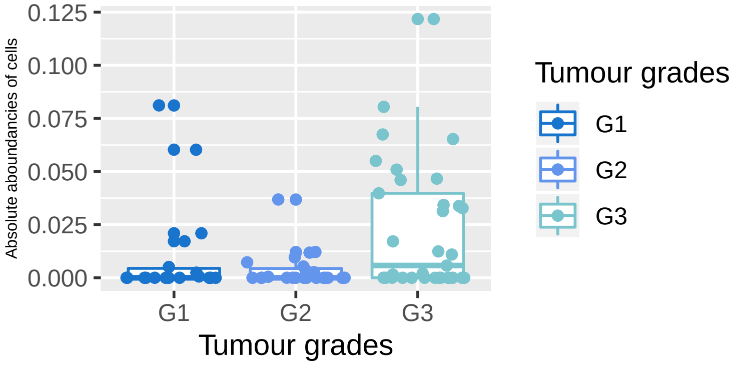

Figure 5: T cells gamma delta across Grades

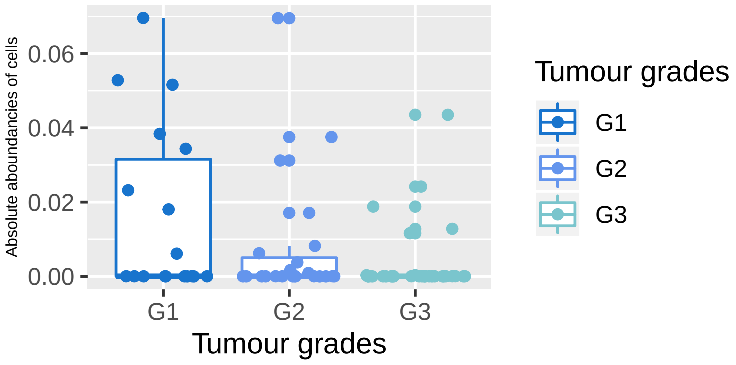

Figure 6: NK cells resting across Grades

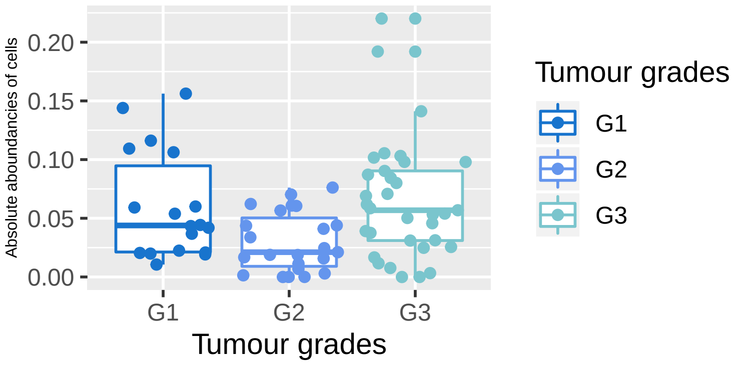

Figure 7: Macrophages M2 across Grades

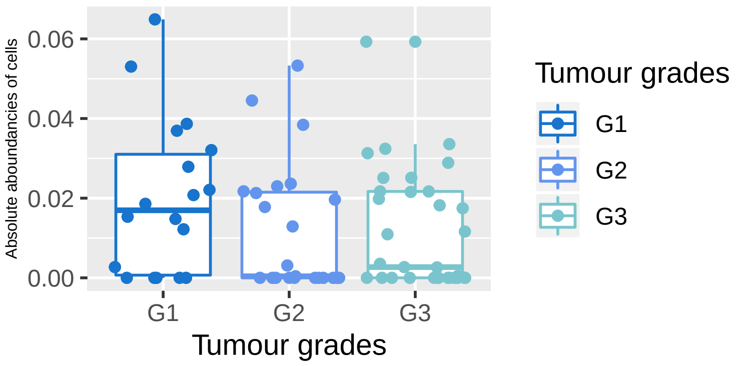

Figure 8: T cells regulatory (Tregs) across Grades

Survival associations

Table 3: Cox Proportional Hazard model of cell type aboundancies with tumour grades and histotypes

CellType	Covar	HR	coeff p.value	logRankTest p.value
B cells memory	Absolute_Proportion	4.0599065	0.7686117	0.2304363
B cells memory	HistotypeEnd	-0.7778097	0.2293032	0.2304363

Table 3: Cox Proportional Hazard model of cell type aboundancies with tumour grades and histotypes *(continued)*

CellType	Covar	HR	coeff p.value	logRankTest p.value
B cells memory	HistotypeMuc	-1.2534063	0.1486161	0.2304363
B cells memory	HistotypeSerHigh	-0.8826044	0.2191923	0.2304363
B cells memory	HistotypeSerLow	-19.0692930	0.9977576	0.2304363
B cells naive	Absolute_Proportion	5.2500149	0.5222174	0.2090989
B cells naive	HistotypeEnd	-0.7405518	0.2553145	0.2090989
B cells naive	HistotypeMuc	-1.1274382	0.1711688	0.2090989
B cells naive	HistotypeSerHigh	-0.8274279	0.2440745	0.2090989
B cells naive	HistotypeSerLow	-18.9416536	0.9978112	0.2090989
Dendritic cells activated	Absolute_Proportion	1.6492173	0.8373994	0.2336555
Dendritic cells activated	HistotypeEnd	-0.7657498	0.2374384	0.2336555
Dendritic cells activated	HistotypeMuc	-1.1634198	0.1559270	0.2336555
Dendritic cells activated	HistotypeSerHigh	-0.8670845	0.2240597	0.2336555
Dendritic cells activated	HistotypeSerLow	-19.0256474	0.9977627	0.2336555
Dendritic cells resting	Absolute_Proportion	-12.9570998	0.6982439	0.2234489
Dendritic cells resting	HistotypeEnd	-0.7592520	0.2409599	0.2234489
Dendritic cells resting	HistotypeMuc	-1.1910037	0.1460947	0.2234489
Dendritic cells resting	HistotypeSerHigh	-0.8482736	0.2311244	0.2234489
Dendritic cells resting	HistotypeSerLow	-19.0368242	0.9977671	0.2234489
Eosinophils	Absolute_Proportion	-4.6382340	0.8373293	0.2334535
Eosinophils	HistotypeEnd	-0.7709391	0.2332976	0.2334535
Eosinophils	HistotypeMuc	-1.1728690	0.1518746	0.2334535
Eosinophils	HistotypeSerHigh	-0.8662574	0.2237242	0.2334535
Eosinophils	HistotypeSerLow	-19.0322072	0.9977641	0.2334535
Macrophages M0	Absolute_Proportion	0.8219196	0.7873982	0.2313370
Macrophages M0	HistotypeEnd	-0.7960112	0.2220880	0.2313370
Macrophages M0	HistotypeMuc	-1.1823497	0.1488906	0.2313370
Macrophages M0	HistotypeSerHigh	-0.8672965	0.2223404	0.2313370
Macrophages M0	HistotypeSerLow	-19.0873307	0.9977489	0.2313370
Macrophages M1	Absolute_Proportion	-71.9809908	0.4910997	0.1936063
Macrophages M1	HistotypeEnd	-0.8667953	0.1858548	0.1936063
Macrophages M1	HistotypeMuc	-1.2551482	0.1275749	0.1936063
Macrophages M1	HistotypeSerHigh	-0.7716658	0.2780625	0.1936063
Macrophages M1	HistotypeSerLow	-18.9669959	0.9977305	0.1936063
Macrophages M2	Absolute_Proportion	-8.5235423	0.2062639	0.1293809
Macrophages M2	HistotypeEnd	-1.1387655	0.0972862	0.1293809
Macrophages M2	HistotypeMuc	-1.3632033	0.0992393	0.1293809
Macrophages M2	HistotypeSerHigh	-1.2153042	0.1021630	0.1293809
Macrophages M2	HistotypeSerLow	-19.2635664	0.9977567	0.1293809
Mast cells activated	Absolute_Proportion	-5.7143618	0.4864330	0.1957252
Mast cells activated	HistotypeEnd	-0.8065803	0.2125082	0.1957252
Mast cells activated	HistotypeMuc	-1.2061584	0.1408280	0.1957252
Mast cells activated	HistotypeSerHigh	-0.9439566	0.1870078	0.1957252
Mast cells activated	HistotypeSerLow	-19.1724933	0.9977812	0.1957252
Mast cells resting	Absolute_Proportion	-41.7676260	0.1303874	0.0677617
Mast cells resting	HistotypeEnd	-0.8033895	0.2151438	0.0677617
Mast cells resting	HistotypeMuc	-1.2757415	0.1208470	0.0677617
Mast cells resting	HistotypeSerHigh	-0.6785027	0.3457662	0.0677617
Mast cells resting	HistotypeSerLow	-18.6881667	0.9978161	0.0677617
Monocytes	Absolute_Proportion	-17.8568169	0.4939923	0.1984225
Monocytes	HistotypeEnd	-0.8447799	0.1939263	0.1984225
Monocytes	HistotypeMuc	-1.3020648	0.1179779	0.1984225
Monocytes	HistotypeSerHigh	-1.0064056	0.1692282	0.1984225
Monocytes	HistotypeSerLow	-19.1060679	0.9977621	0.1984225
Neutrophils	Absolute_Proportion	16.4797212	0.1203150	0.1293956
Neutrophils	HistotypeEnd	-0.5760799	0.3973954	0.1293956
Neutrophils	HistotypeMuc	-1.0009958	0.2343976	0.1293956
Neutrophils	HistotypeSerHigh	-0.6699476	0.3626197	0.1293956
Neutrophils	HistotypeSerLow	-19.2939124	0.9981812	0.1293956
NK cells activated	Absolute_Proportion	-5.1250705	0.6086266	0.2162272
NK cells activated	HistotypeEnd	-0.8569154	0.1962952	0.2162272
NK cells activated	HistotypeMuc	-1.2836881	0.1270479	0.2162272
NK cells activated	HistotypeSerHigh	-0.8902134	0.2105973	0.2162272
NK cells activated	HistotypeSerLow	-19.0247794	0.9977686	0.2162272
NK cells resting	Absolute_Proportion	-17.4221837	0.4376191	0.1844262
NK cells resting	HistotypeEnd	-0.7629253	0.2380843	0.1844262

Table 3: Cox Proportional Hazard model of cell type aboundancies with tumour grades and histotypes *(continued)*

CellType	Covar	HR	coeff p.value	logRankTest p.value
NK cells resting	HistotypeMuc	-0.9902124	0.2364095	0.1844262
NK cells resting	HistotypeSerHigh	-0.9243841	0.1941853	0.1844262
NK cells resting	HistotypeSerLow	-19.0299743	0.9977679	0.1844262
Plasma cells	Absolute_Proportion	-8.6025193	0.5593424	0.2083414
Plasma cells	HistotypeEnd	-0.7820597	0.2270257	0.2083414
Plasma cells	HistotypeMuc	-1.2379589	0.1337926	0.2083414
Plasma cells	HistotypeSerHigh	-0.8599276	0.2250133	0.2083414
Plasma cells	HistotypeSerLow	-19.0788581	0.9977623	0.2083414
T cells CD4 memory activated	Absolute_Proportion	-6.4256843	0.4244377	0.1779390
T cells CD4 memory activated	HistotypeEnd	-0.7766263	0.2300005	0.1779390
T cells CD4 memory activated	HistotypeMuc	-1.2663362	0.1240207	0.1779390
T cells CD4 memory activated	HistotypeSerHigh	-0.7955520	0.2625910	0.1779390
T cells CD4 memory activated	HistotypeSerLow	-19.1386910	0.9977746	0.1779390
T cells CD4 memory resting	Absolute_Proportion	4.2238762	0.5031878	0.2048764
T cells CD4 memory resting	HistotypeEnd	-0.8583449	0.1941471	0.2048764
T cells CD4 memory resting	HistotypeMuc	-1.2903158	0.1245841	0.2048764
T cells CD4 memory resting	HistotypeSerHigh	-0.8449533	0.2329934	0.2048764
T cells CD4 memory resting	HistotypeSerLow	-19.1869860	0.9977327	0.2048764
T cells CD4 naive	Absolute_Proportion	-7.5017351	0.6533815	0.2196317
T cells CD4 naive	HistotypeEnd	-0.7888535	0.2229211	0.2196317
T cells CD4 naive	HistotypeMuc	-1.1495979	0.1603867	0.2196317
T cells CD4 naive	HistotypeSerHigh	-0.8676981	0.2207797	0.2196317
T cells CD4 naive	HistotypeSerLow	-19.0194235	0.9977678	0.2196317
T cells CD8	Absolute_Proportion	-25.8800039	0.1311778	0.0650927
T cells CD8	HistotypeEnd	-1.0153306	0.1191768	0.0650927
T cells CD8	HistotypeMuc	-1.4167435	0.0856405	0.0650927
T cells CD8	HistotypeSerHigh	-1.0335494	0.1480904	0.0650927
T cells CD8	HistotypeSerLow	-19.5068443	0.9978389	0.0650927
T cells follicular helper	Absolute_Proportion	-5.6367123	0.6494264	0.2195310
T cells follicular helper	HistotypeEnd	-0.8119168	0.2118280	0.2195310
T cells follicular helper	HistotypeMuc	-1.2158479	0.1391512	0.2195310
T cells follicular helper	HistotypeSerHigh	-0.8502165	0.2303207	0.2195310
T cells follicular helper	HistotypeSerLow	-18.9618864	0.9977734	0.2195310
T cells gamma delta	Absolute_Proportion	-16.8816913	0.2798335	0.1412001
T cells gamma delta	HistotypeEnd	-0.9026658	0.1661073	0.1412001
T cells gamma delta	HistotypeMuc	-1.2416238	0.1298938	0.1412001
T cells gamma delta	HistotypeSerHigh	-0.6744749	0.3490898	0.1412001
T cells gamma delta	HistotypeSerLow	-19.0737160	0.9977514	0.1412001
T cells regulatory (Tregs)	Absolute_Proportion	16.7850638	0.3191202	0.1727992
T cells regulatory (Tregs)	HistotypeEnd	-0.8486342	0.1953066	0.1727992
T cells regulatory (Tregs)	HistotypeMuc	-1.1991514	0.1432380	0.1727992
T cells regulatory (Tregs)	HistotypeSerHigh	-0.9394073	0.1884476	0.1727992
T cells regulatory (Tregs)	HistotypeSerLow	-19.3744426	0.9976909	0.1727992

Anova test with the total amount of immune cells for patient

Table 4: Anova results of total amount of immune cells across grades and subtypes

clin	comp	diff	lwr	upr	p_anova	p_adj
Grade	G2-G1	-0.10491	-0.23028	0.02047	0.0452	0.11890
Grade	G3-G1	0.00218	-0.11456	0.11892	0.0452	0.99890
Grade	G3-G2	0.10709	-0.00113	0.21531	0.0452	0.05306
Histotype	End-Cc	-0.08040	-0.24229	0.08149	0.4107	0.63591
Histotype	Muc-Cc	-0.07022	-0.23641	0.09597	0.4107	0.76118
Histotype	SerHigh-Cc	-0.04573	-0.21441	0.12296	0.4107	0.94143
Histotype	SerLow-Cc	0.03450	-0.17210	0.24110	0.4107	0.99000
Histotype	Muc-End	0.01018	-0.14910	0.16947	0.4107	0.99976
Histotype	SerHigh-End	0.03468	-0.12721	0.19657	0.4107	0.97471
Histotype	SerLow-End	0.11490	-0.08618	0.31599	0.4107	0.50271
Histotype	SerHigh-Muc	0.02449	-0.14169	0.19068	0.4107	0.99379
Histotype	SerLow-Muc	0.10472	-0.09984	0.30928	0.4107	0.60876
Histotype	SerLow-SerHigh	0.08023	-0.12637	0.28682	0.4107	0.81265
Relapse	Y-N	-0.01192	-0.09960	0.07576	0.7872	0.78723

Survival association with the total amount of immune cells for patient

Table 5: Cox Proportional Hazard model of immune cell presence within tumour grades and histotypes

Covar	coef	exp.coef.	se.coef.	z	Pr...z..	Logtest
sumByPat	-2.3198253	0.0982908	1.9421292	-1.1944752	0.2322921	0.1382812
HistotypeEnd	-0.9307995	0.3942384	0.6543936	-1.4223848	0.1549146	0.1382812
HistotypeMuc	-1.2830123	0.2772010	0.8204951	-1.5637051	0.1178869	0.1382812
HistotypeSerHigh	-0.9789405	0.3757089	0.7152916	-1.3685894	0.1711276	0.1382812
HistotypeSerLow	-18.9807844	0.0000000	6613.8110218	-0.0028699	0.9977102	0.1382812
