# Supplementary material for: Transcriptional Characterization of Stage I Epithelial Ovarian Cancer: A Multicentric Study
Source: Cells. 2019 Dec 1;8(12):1554. doi: 10.3390/cells8121554 (PMC6952972; doi:10.3390/cells8121554)
Supplement: Supplementary file 1 [file cells-08-01554-s001.zip › SuppMat3.pdf]

Supplementary Material 3: immunophenoscore of stage I EOC

Contents

IPS differences across clinical annotations . . . 1
By Subtypes . . . 1
By Relapse status . . . 2
MHC differences across clinical annotations . . . 2
By Subtypes . . . 2
By Relapse status . . . 2
EC differences across clinical annotations . . . 2
By Subtypes . . . 2
By Relapse status . . . 3
SC differences across clinical annotations . . . 3
By Subtypes . . . 3
By Relapse status . . . 3
CP differences across clinical annotations . . . 4
By Subtypes . . . 4
By Relapse status . . . 4
IPS, MHC, CP, EC, SC by grades . . . 5
Progression free survival of patients with different IPS . . . 6
Clear cell histotype . . . 7
Mucinous histotype . . . 7
Serous High Grade histotype . . . 8
Serous Low Grade histotype . . . 9
Endometrioid histotype . . . 10

IPS differences across clinical annotations

By Subtypes

	diff	lwr	upr	p adj
End-Cc	-0.3684211	-1.7433471	1.006505	0.9437329
Muc-Cc	0.2352941	-1.1761205	1.646709	0.9900686
SerHigh-Cc	0.3125000	-1.1201401	1.745140	0.9729765
SerLow-Cc	0.3750000	-1.3796186	2.129619	0.9749176
Muc-End	0.6037152	-0.7490801	1.956511	0.7226909
SerHigh-End	0.6809211	-0.6940049	2.055847	0.6383947
SerLow-End	0.7434211	-0.9643993	2.451241	0.7407110

	diff	lwr	upr	p adj
SerHigh-Muc	0.0772059	-1.3342087	1.488620	0.9998734
SerLow-Muc	0.1397059	-1.5976254	1.877037	0.9994168
SerLow-SerHigh	0.0625000	-1.6921186	1.817119	0.9999771

By Relapse status

	diff	lwr	upr	p adj
Y-N	0.0406926	-0.6981925	0.7795778	0.9129163

MHC differences across clinical annotations

By Subtypes

	diff	lwr	upr	p adj
End-Cc	-0.0271968	-0.4159145	0.3615209	0.9996637
Muc-Cc	-0.0225766	-0.4216103	0.3764571	0.9998553
SerHigh-Cc	0.1361530	-0.2688816	0.5411876	0.8798980
SerLow-Cc	0.0234626	-0.4726014	0.5195267	0.9999291
Muc-End	0.0046202	-0.3778407	0.3870811	0.9999997
SerHigh-End	0.1633498	-0.2253679	0.5520675	0.7647925
SerLow-End	0.0506594	-0.4321738	0.5334927	0.9983389
SerHigh-Muc	0.1587296	-0.2403042	0.5577633	0.7989403
SerLow-Muc	0.0460392	-0.4451374	0.5372158	0.9989329
SerLow-SerHigh	-0.1126904	-0.6087544	0.3833737	0.9686835

By Relapse status

	diff	lwr	upr	p adj
Y-N	-0.0988096	-0.304978	0.1073588	0.3427076

EC differences across clinical annotations

By Subtypes

	diff	lwr	upr	p adj
End-Cc	-0.0737779	-0.2099749	0.0624191	0.5555096

	diff	lwr	upr	p adj
Muc-Cc	-0.1118349	-0.2516464	0.0279765	0.1775305
SerHigh-Cc	0.0365554	-0.1053586	0.1784694	0.9509781
SerLow-Cc	-0.0270295	-0.2008380	0.1467789	0.9923758
Muc-End	-0.0380571	-0.1720618	0.0959477	0.9312603
SerHigh-End	0.1103333	-0.0258637	0.2465303	0.1676757
SerLow-End	0.0467483	-0.1224244	0.2159211	0.9374120
SerHigh-Muc	0.1483904	0.0085789	0.2882018	0.0320143
SerLow-Muc	0.0848054	-0.0872906	0.2569014	0.6427434
SerLow-SerHigh	-0.0635850	-0.2373934	0.1102235	0.8434612

By Relapse status

	diff	lwr	upr	p adj
Y-N	0.0214501	-0.0556312	0.0985314	0.5809201

SC differences across clinical annotations

By Subtypes

	diff	lwr	upr	p adj
End-Cc	0.1146105	-0.1002654	0.3294865	0.5703923
Muc-Cc	0.1136760	-0.1069024	0.3342545	0.6026361
SerHigh-Cc	0.1667009	-0.0571947	0.3905965	0.2383699
SerLow-Cc	0.1405098	-0.1337052	0.4147248	0.6078961
Muc-End	-0.0009345	-0.2123518	0.2104828	1.0000000
SerHigh-End	0.0520904	-0.1627856	0.2669663	0.9604527
SerLow-End	0.0258992	-0.2410020	0.2928005	0.9987772
SerHigh-Muc	0.0530249	-0.1675536	0.2736034	0.9616221
SerLow-Muc	0.0268338	-0.2446795	0.2983471	0.9986861
SerLow-SerHigh	-0.0261911	-0.3004061	0.2480239	0.9988510

By Relapse status

	diff	lwr	upr	p adj
Y-N	0.0222707	-0.09472	0.1392613	0.705547

CP differences across clinical annotations

By Subtypes

	diff	lwr	upr	p adj
End-Cc	-0.0970221	-0.3834508	0.1894065	0.8769328
Muc-Cc	0.1106692	-0.1833608	0.4046992	0.8293310
SerHigh-Cc	-0.1913086	-0.4897604	0.1071432	0.3849951
SerLow-Cc	-0.0092001	-0.3747274	0.3563271	0.9999943
Muc-End	0.2076913	-0.0741269	0.4895096	0.2476803
SerHigh-End	-0.0942864	-0.3807151	0.1921422	0.8877166
SerLow-End	0.0878220	-0.2679561	0.4436001	0.9578247
SerHigh-Muc	-0.3019778	-0.5960078	-0.0079478	0.0412103
SerLow-Muc	-0.1198694	-0.4817953	0.2420566	0.8854821
SerLow-SerHigh	0.1821084	-0.1834188	0.5476357	0.6331473

By Relapse status

	diff	lwr	upr	p adj
Y-N	0.0784749	-0.0810774	0.2380272	0.3302689

IPS, MHC, CP, EC, SC by grades

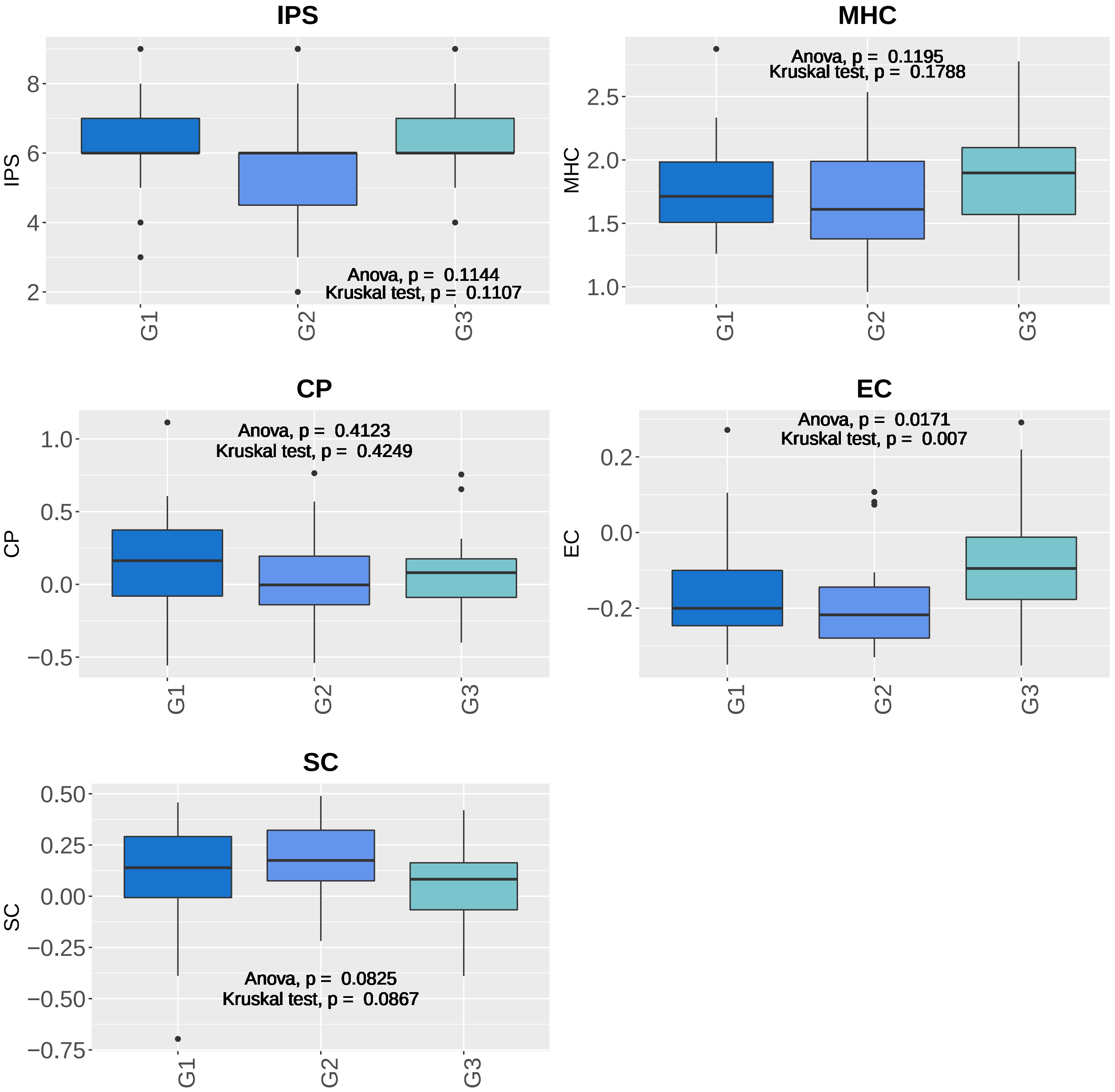

Progression free survival of patients with different IPS

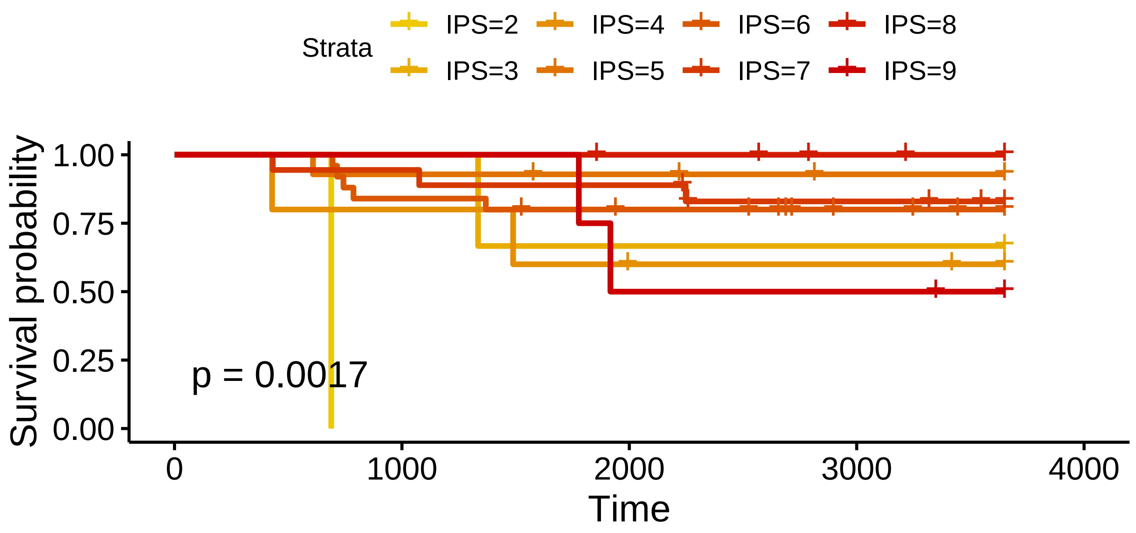

Figure 1: Survival of patients divided by IPS

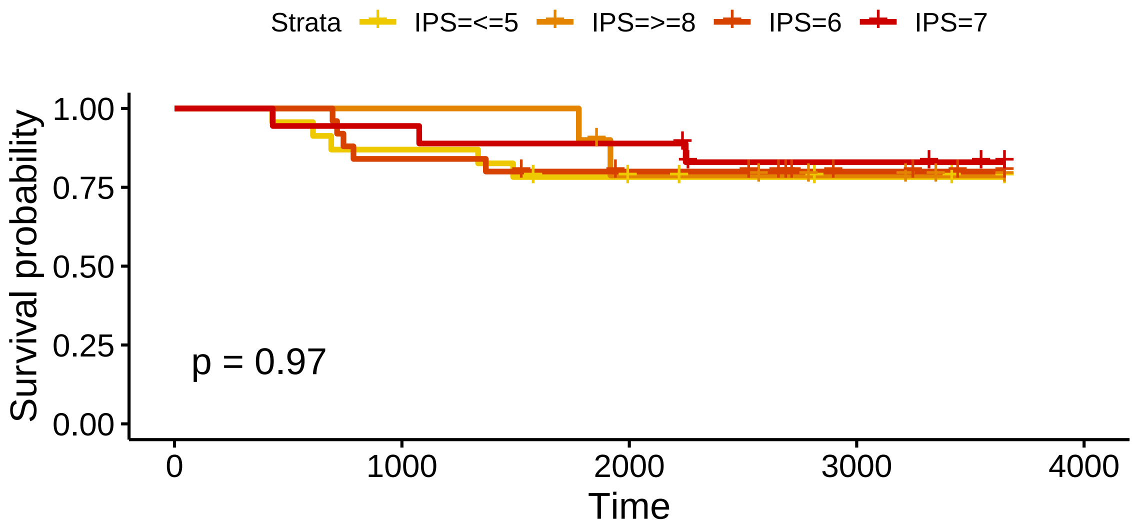

Figure 2: Frequencies of patients divided by IPS aggregating less frequent and extreme occurrencies

Clear cell histotype

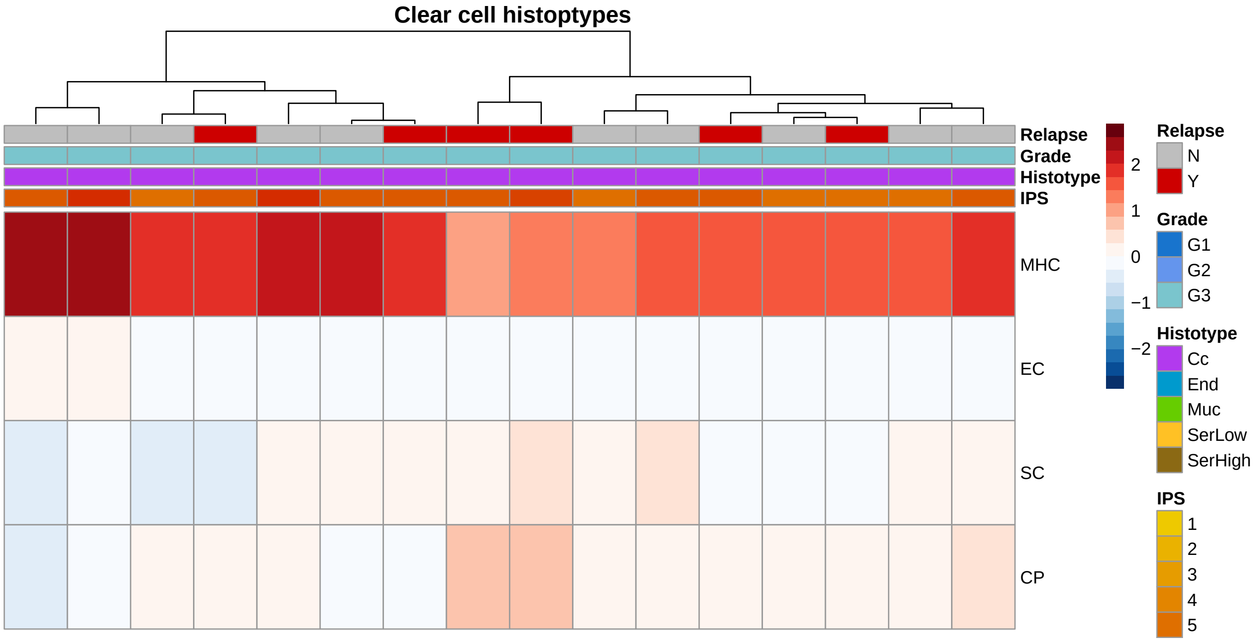

	diff	lwr	upr	p adj
Y-N	0	-1.107563	1.107563	1

Mucinous histotype

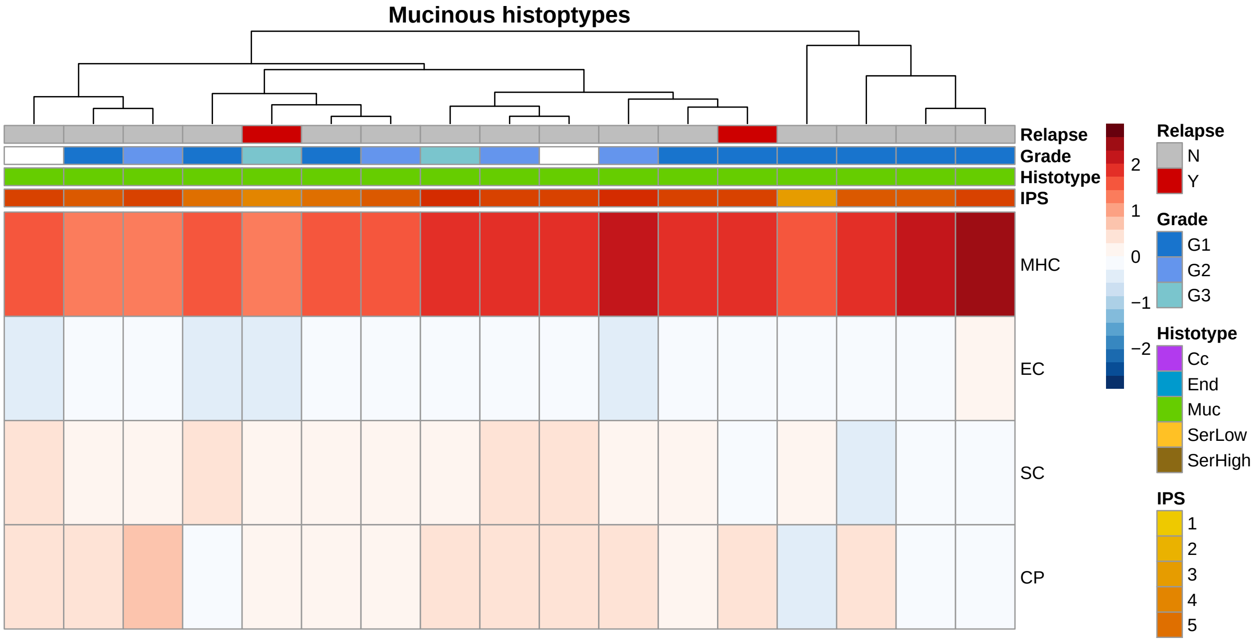

	diff	lwr	upr	p adj
Y-N	-0.8333333	-3.018959	1.352293	0.4291137

Serous High Grade histotype

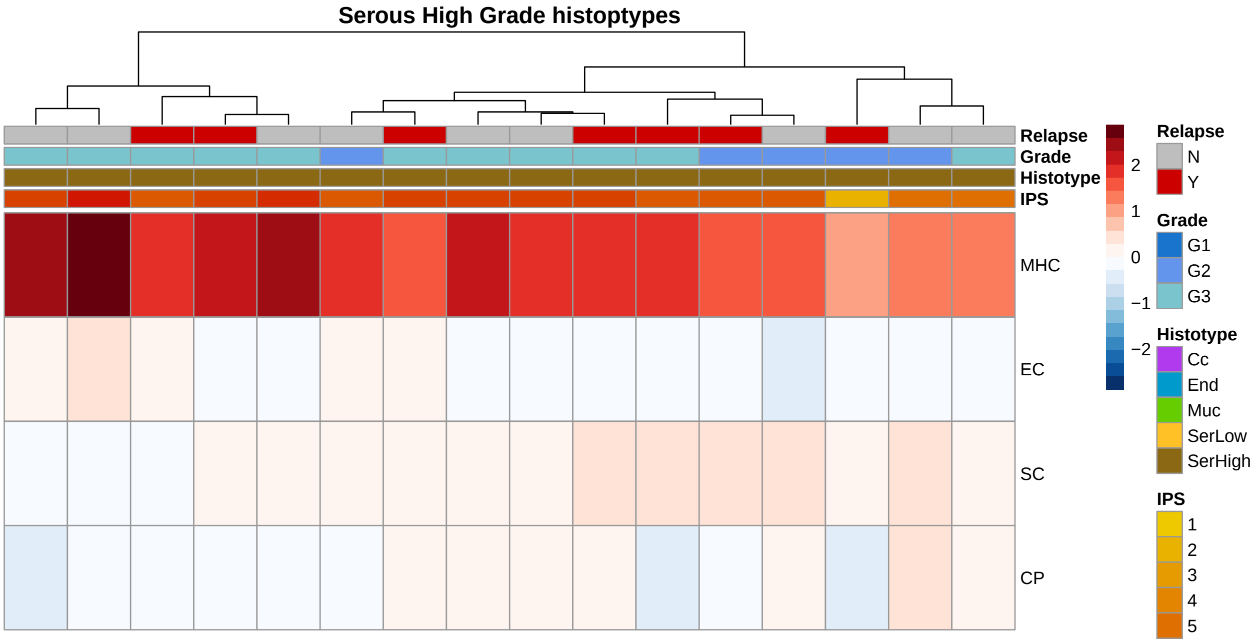

	diff	lwr	upr	p adj
Y-N	-0.8095238	-2.465388	0.8463403	0.3121505

Serous Low Grade histotype

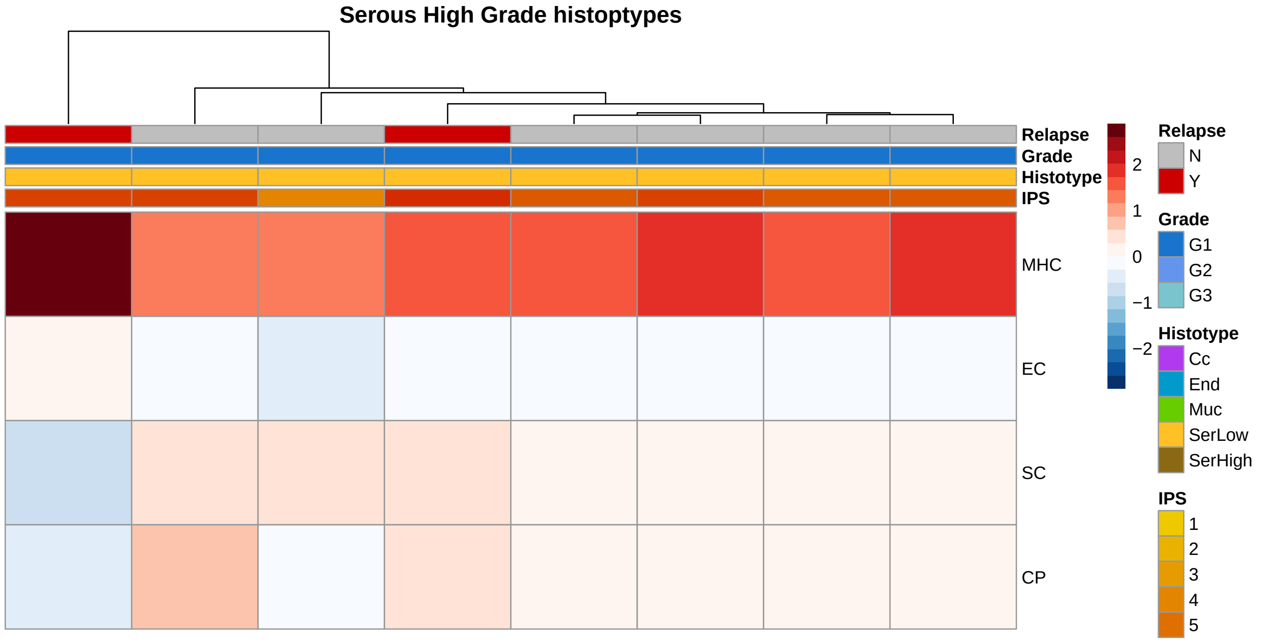

	diff	lwr	upr	p adj
Y-N	1.5	-0.5794751	3.579475	0.1279986

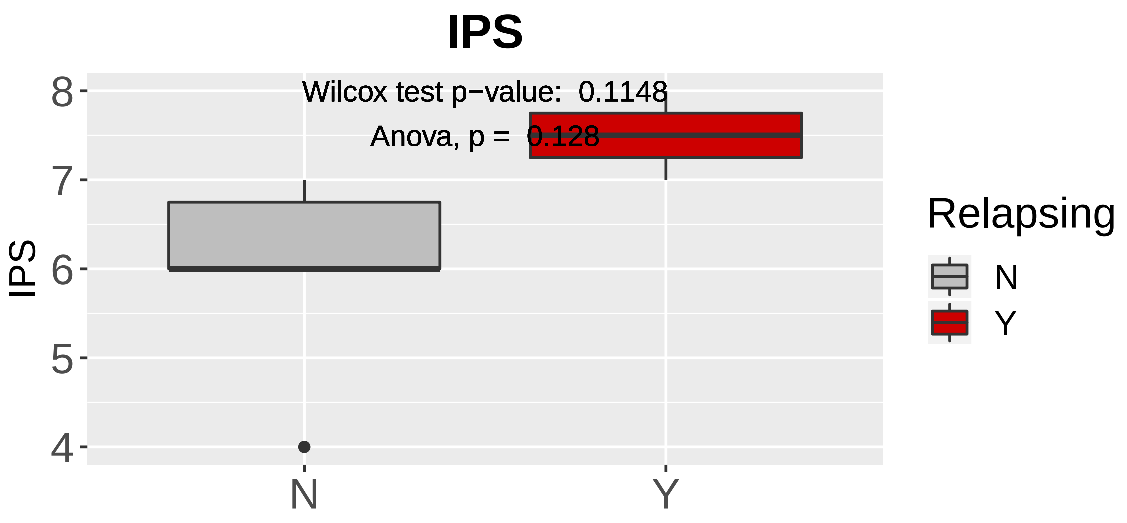

Figure 3: IPS plot of low grade serous patients divided by tumor relapse

Endometrioid histotype

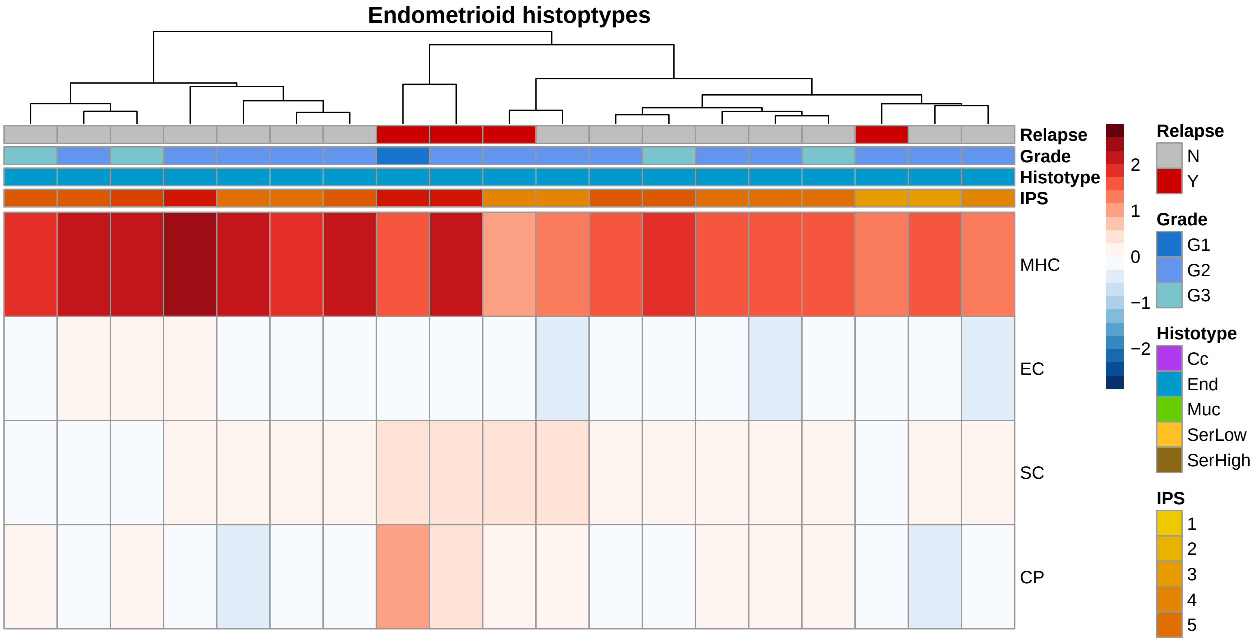

	diff	lwr	upr	p adj
Y-N	0.7833333	-1.418768	2.985434	0.4632125
