# Supplementary material for: Transcriptional Characterization of Stage I Epithelial Ovarian Cancer: A Multicentric Study
Source: Cells. 2019 Dec 1;8(12):1554. doi: 10.3390/cells8121554 (PMC6952972; doi:10.3390/cells8121554)
Supplement: Supplementary file 1 [file cells-08-01554-s001.zip › SuppMat4df.pdf]

Supplementary File 4: micrographite analyses of gene and miRNA expression data of histotype circuit

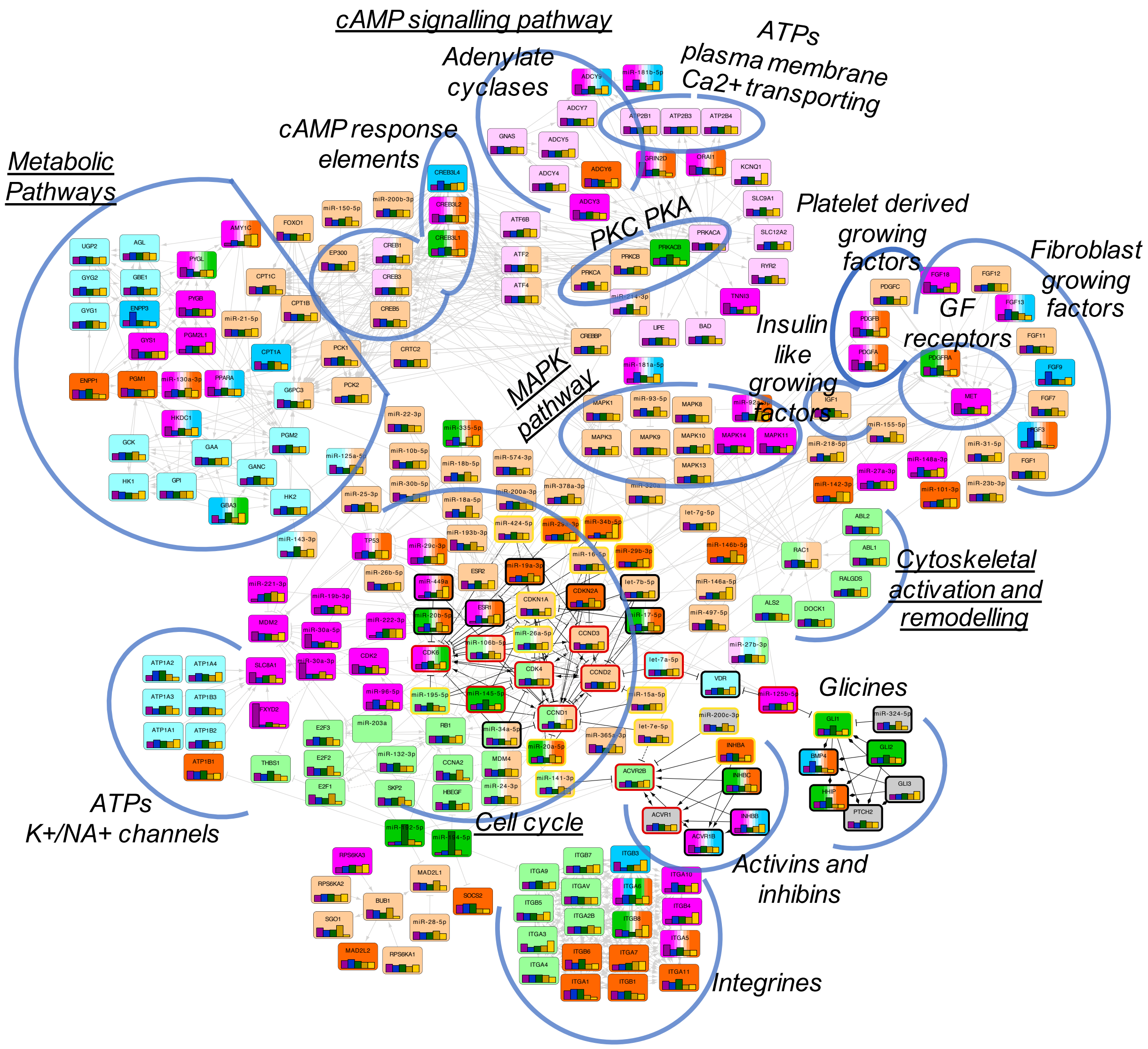

Figure 1: Histotype circuit

Circuit legend

Nodes have been colored by differential expressions, pink for clear cell, green for mucinous, blu for endometrioid and brown for high-grade serous and yellow for low-grade serous. Non-DEGs were colored according to the micrographite analysis they came from, using a lighter shade of the histotype colors.

Genes that came from more than one path or differentially expressed in more than one histotype were colored using a multicolor gradient.

The prognostic circuit was added to the final path. Genes not already present in the histotype analyses were colored in gray.

Histograms were plotted inside nodes: each bar corresponds to the mean expression value in one histotype normalized by the mean expression value across all histotypes. In order to emphasize the differences between histotypes, means were not computed using log2 expression data, but non log2-trasformed data was used instead. Histogram bars were colored using a darker shade of the histotype colors

Nodes belonging to the prognostic circuit were given a thicker border, which was colored red if the gene was upregulated in the prognostic circuit, yellow if it was downregulated and black as default following Calura et al. 2016. Edges belonging to the prognostic circuit were colored black otherwise are gray.
