# Supplementary material for: Transcriptional Characterization of Stage I Epithelial Ovarian Cancer: A Multicentric Study
Source: Cells. 2019 Dec 1;8(12):1554. doi: 10.3390/cells8121554 (PMC6952972; doi:10.3390/cells8121554)
Supplement: Supplementary file 1 [file cells-08-01554-s001.zip › SuppMat5.pdf]

Supplementary File 5: Analyses of qRT-PCR data across histoptypes

Contents

Pairwise t-test of gene qRT-PCR expression values across histotypes . . . 2
Training set . . . 2
Validation set . . . 3
Concordant results training and validation set . . . 5
Boxplots of qRT-PCR expression values across histotypes . . . 6
Training set . . . 6
Tables of average values of qRT-PCR expression across histotypes . . . 7
Training set . . . 7
Validation set . . . 8

Pairwise t-test of gene qRT-PCR expression values across histotypes

Training set

Table 1: t-test p-value and adjusted p-value of miRNA qRT-PCR values of training set samples

miRNAs	Muc vs SerHigh		Muc vs SerLow		Muc vs End		Muc vs Cc		SerHigh vs End		SerLow vs End		SerHigh vs Cc		SerLow vs Cc		End vs Cc		SerH vs SerL		Muc vs not Muc		SerHigh vs not Ser		SerLow vs not Ser		End vs not End		Cc vs not Cc	
	p	adj-p	p	adj-p	p	adj-p	p	adj-p	p	adj-p	p	adj-p	p	adj-p	p	adj-p	p	adj-p	p	adj-p	p	adj-p	p	adj-p	p	adj-p	p	adj-p	p	adj-p
hsa-let-7a-5p	0.0012	0.0091	0.9849	0.9863	0.0602	0.3635	0.0016	0.0074	0.0520	0.2758	0.1683	0.5946	0.4018	0.6980	0.0169	0.0805	0.1688	0.5215	0.0221	0.2328	0.0046	0.0351	0.0029	0.0520	0.0952	0.3789	0.7149	0.9402	0.1506	0.3471
hsa-let-7b-5p	0.6109	0.8015	0.4295	0.9118	0.2764	0.6668	0.0939	0.2205	0.5474	0.9218	0.9402	0.9616	0.2105	0.6562	0.5474	0.7077	0.4770	0.7506	0.6886	0.8307	0.1750	0.3866	0.5577	0.8445	0.9121	0.9478	0.7145	0.9402	0.1282	0.3397
hsa-let-7e-5p	0.3293	0.6301	0.4429	0.9118	0.8488	0.9572	0.1694	0.3453	0.3732	0.8280	0.4744	0.8940	0.6283	0.8763	0.7169	0.8443	0.1801	0.5215	0.9991	0.9991	0.2802	0.4950	0.6248	0.8715	0.7729	0.8716	0.3804	0.8400	0.1827	0.4034
hsa-miR-106b-5p	0.9634	0.9881	0.6550	0.9118	0.9684	0.9684	0.5031	0.7207	0.9245	0.9796	0.6225	0.8940	0.4476	0.7413	0.8794	0.9322	0.4627	0.7506	0.4847	0.7784	0.7177	0.8766	0.7791	0.8976	0.7269	0.8376	0.6424	0.9402	0.3697	0.6088
hsa-miR-125b-5p	0.3793	0.6445	0.1973	0.9118	0.1810	0.6101	0.0707	0.1784	0.0412	0.2671	0.0593	0.4199	0.0233	0.1375	0.0675	0.1871	0.3724	0.7506	0.4539	0.7784	0.4524	0.6480	0.0991	0.2627	0.1020	0.3789	0.3161	0.7970	0.0098	0.0528
hsa-miR-141-3p	0.4724	0.6767	0.5296	0.9118	0.1842	0.6101	0.9267	0.9446	0.0207	0.1910	0.8003	0.9426	0.3075	0.6831	0.4259	0.6091	0.0949	0.4245	0.2133	0.5652	0.6689	0.8732	0.0789	0.2323	0.4667	0.7216	0.0608	0.4231	0.7881	0.8887
hsa-miR-145-5p	0.6931	0.8543	0.0377	0.3874	0.2476	0.6668	0.4655	0.7050	0.1149	0.4781	0.0068	0.1199	0.2810	0.6831	0.0349	0.1323	0.7910	0.8556	0.0468	0.2337	0.8235	0.8908	0.4454	0.7232	0.0126	0.1676	0.1005	0.5326	0.3110	0.5510
hsa-miR-15a-5p	0.3891	0.6445	0.4455	0.9118	0.5446	0.8121	0.4636	0.7050	0.7806	0.9218	0.2279	0.7549	0.8953	0.9481	0.1872	0.3908	0.8856	0.9150	0.1650	0.4999	0.5047	0.7024	0.4522	0.7232	0.1825	0.4396	0.7473	0.9402	0.6086	0.8147
hsa-miR-16-5p	0.0499	0.1652	0.9723	0.9863	0.9475	0.9684	0.2145	0.4061	0.0182	0.1910	0.9232	0.9616	0.3361	0.6980	0.3688	0.5585	0.1041	0.4245	0.1698	0.4999	0.2289	0.4377	0.0142	0.1530	0.5054	0.7216	0.1645	0.5812	0.4795	0.7059
hsa-miR-17-5p	0.0261	0.1062	0.5272	0.9118	0.4791	0.8054	0.1043	0.2303	0.1316	0.4984	0.3174	0.8940	0.3666	0.6980	0.0706	0.1871	0.4630	0.7506	0.0467	0.2337	0.1434	0.3763	0.0199	0.1530	0.1342	0.3952	0.6633	0.9402	0.4940	0.7076
hsa-miR-192-5p	0.0000	0.0000	0.0000	0.0000	0.0000	0.0000	0.0000	0.0000	0.6842	0.9218	0.8806	0.9527	0.1767	0.6247	0.7796	0.8982	0.6812	0.8535	0.3771	0.7724	0.0000	0.0000	0.0484	0.1972	0.1462	0.3952	0.0130	0.2472	0.0110	0.0528
hsa-miR-194-5p	0.0000	0.0000	0.0000	0.0000	0.0000	0.0000	0.0000	0.0000	0.8083	0.9218	0.7085	0.8940	0.1768	0.6247	0.9347	0.9527	0.5082	0.7506	0.4415	0.7784	0.0000	0.0000	0.0643	0.2263	0.1312	0.3952	0.0260	0.2798	0.0109	0.0528
hsa-miR-195-5p	0.0191	0.0843	0.7704	0.9863	0.1304	0.5486	0.0118	0.0418	0.2081	0.6488	0.0970	0.4751	0.8854	0.9481	0.0173	0.0805	0.1448	0.4796	0.0308	0.2328	0.0191	0.1015	0.0781	0.2323	0.0858	0.3789	0.9016	0.9498	0.0535	0.1891
hsa-miR-19a-3p	0.7777	0.9331	0.4494	0.9118	0.9651	0.9684	0.4246	0.6819	0.8093	0.9218	0.4759	0.8940	0.3093	0.6831	0.1917	0.3908	0.4018	0.7506	0.6249	0.8280	0.9966	0.9966	0.6458	0.8776	0.3554	0.6481	0.9470	0.9510	0.2124	0.4330
hsa-miR-200c-3p	0.9939	0.9939	0.6444	0.9118	0.6424	0.8959	0.9116	0.9446	0.5141	0.9218	0.6247	0.8940	0.8664	0.9481	0.2484	0.4267	0.2127	0.5367	0.5113	0.7784	0.7659	0.8766	0.7790	0.8976	0.5101	0.7216	0.5544	0.9326	0.6149	0.8147
hsa-miR-20a-5p	0.4544	0.6767	0.5686	0.9118	0.8771	0.9684	0.5647	0.7483	0.6315	0.9218	0.6594	0.8940	0.8480	0.9481	0.4122	0.6069	0.7568	0.8535	0.3789	0.7724	0.6755	0.8732	0.4639	0.7232	0.4827	0.7216	0.8526	0.9415	0.7077	0.8320
hsa-miR-20b-5p	0.6713	0.8471	0.8194	0.9863	0.5516	0.8121	0.8240	0.9446	0.8174	0.9218	0.5471	0.8940	0.4892	0.7857	0.9169	0.9527	0.4079	0.7506	0.6049	0.8246	0.7773	0.8766	0.7330	0.8829	0.6261	0.7862	0.4098	0.8513	0.5544	0.7732
hsa-miR-214-3p	0.0000	0.0005	0.6570	0.9118	0.0014	0.0153	0.0000	0.0001	0.3073	0.8143	0.0040	0.1054	0.8778	0.9481	0.0000	0.0001	0.2122	0.5367	0.0002	0.0096	0.0000	0.0003	0.0156	0.1530	0.0043	0.0762	0.2981	0.7970	0.0123	0.0544
hsa-miR-26a-5p	0.0072	0.0346	0.9291	0.9863	0.0790	0.4037	0.0033	0.0135	0.3287	0.8280	0.1551	0.5874	0.9999	0.9999	0.0095	0.0630	0.2958	0.6990	0.0283	0.2328	0.0083	0.0488	0.0683	0.2263	0.1025	0.3789	0.8455	0.9415	0.0797	0.2288
hsa-miR-29a-3p	0.1899	0.4575	0.0512	0.3874	0.3262	0.6668	0.0957	0.2205	0.7376	0.9218	0.0132	0.1754	0.7564	0.9481	0.0025	0.0187	0.5136	0.7506	0.0070	0.1189	0.3280	0.5432	0.3008	0.5705	0.0025	0.0652	0.5807	0.9326	0.1472	0.3471
hsa-miR-29b-3p	0.0044	0.0260	0.1692	0.9118	0.0031	0.0274	0.0003	0.0016	0.9426	0.9796	0.0024	0.1054	0.4083	0.6980	0.0007	0.0097	0.4482	0.7506	0.0035	0.0917	0.0069	0.0455	0.1969	0.4743	0.0005	0.0244	0.1364	0.5744	0.0254	0.1034
hsa-miR-29c-3p	0.4439	0.6767	0.9291	0.9863	0.3834	0.7508	0.0049	0.0187	0.7270	0.9218	0.4576	0.8940	0.0019	0.0205	0.0020	0.0176	0.1294	0.4573	0.3732	0.7724	0.1139	0.3551	0.6650	0.8811	0.2937	0.6210	0.8714	0.9426	0.0073	0.0528
hsa-miR-30a-3p	0.8097	0.9331	0.5548	0.9118	0.8213	0.9572	0.0000	0.0000	0.9633	0.9818	0.7056	0.8940	0.0000	0.0000	0.0000	0.0000	0.0000	0.0000	0.4771	0.7784	0.0613	0.2498	0.0426	0.1881	0.1491	0.3952	0.0264	0.2798	0.0000	0.0000
hsa-miR-30a-5p	0.9686	0.9881	0.5980	0.9118	0.7880	0.9492	0.0000	0.0000	0.7934	0.9218	0.6702	0.8940	0.0000	0.0000	0.0000	0.0000	0.0000	0.0000	0.5494	0.8089	0.0354	0.1565	0.0332	0.1599	0.1255	0.3952	0.0140	0.2472	0.0000	0.0000
hsa-miR-324-5p	0.0743	0.2189	0.2435	0.9118	0.4641	0.8054	0.7789	0.9409	0.1751	0.5799	0.4293	0.8940	0.1118	0.4557	0.2936	0.4577	0.6485	0.8535	0.7772	0.8955	0.1952	0.4069	0.0317	0.1599	0.5446	0.7216	0.7402	0.9402	0.3840	0.6088
hsa-miR-34a-5p	0.8099	0.9331	0.2456	0.9118	0.2555	0.6668	0.7874	0.9409	0.1737	0.5799	0.3744	0.8940	0.6063	0.8684	0.2146	0.4006	0.3160	0.6990	0.2087	0.5652	0.5168	0.7024	0.2520	0.5343	0.2375	0.5245	0.2732	0.7970	0.8684	0.9205
hsa-miR-34b-5p	0.2154	0.4964	0.3598	0.9118	0.3271	0.6668	0.7492	0.9409	0.8449	0.9329	0.8808	0.9527	0.1040	0.4557	0.2192	0.4006	0.1870	0.5215	0.9929	0.9991	0.3528	0.5667	0.2934	0.5705	0.5281	0.7216	0.4184	0.8513	0.1505	0.3471
hsa-miR-424-5p	0.3567	0.6301	0.2772	0.9118	0.7378	0.9094	0.7989	0.9409	0.5325	0.9218	0.1551	0.5874	0.5992	0.8684	0.2591	0.4291	0.9809	0.9809	0.0500	0.2337	0.7720	0.8766	0.3014	0.5705	0.0951	0.3789	0.8160	0.9402	0.8656	0.9205
hsa-miR-449a	0.0031	0.0208	0.5105	0.9118	0.6908	0.9094	0.5272	0.7353	0.0066	0.1910	0.6924	0.8940	0.0742	0.3624	0.9600	0.9600	0.7204	0.8535	0.1361	0.4509	0.1491	0.3763	0.0024	0.0520	0.8864	0.9396	0.3038	0.7970	0.7221	0.8320
hsa-miR-96-5p	0.4640	0.6767	0.9863	0.9863	0.5985	0.8572	0.0003	0.0018	0.7916	0.9218	0.6623	0.8940	0.0011	0.0140	0.0020	0.0176	0.0004	0.0058	0.5140	0.7784	0.1014	0.3359	0.7105	0.8829	0.3291	0.6229	0.3708	0.8400	0.0000	0.0002

Table 2: t-test p-value and adjusted p-value of gene qRT-PCR values of training set samples

miRNAs	Muc vs SerHigh		Muc vs SerLow		Muc vs End		Muc vs Cc		SerHigh vs End		SerLow vs End		SerHigh vs Cc		SerLow vs Cc		End vs Cc		SerH vs SerL		Muc vs not Muc		SerHigh vs not Ser		SerLow vs not Ser		End vs not End		Cc vs not Cc	
	p	adj-p	p	adj-p	p	adj-p	p	adj-p	p	adj-p	p	adj-p	p	adj-p	p	adj-p	p	adj-p	p	adj-p	p	adj-p	p	adj-p	p	adj-p	p	adj-p	p	adj-p
ACVR1	0.8575	0.9468	0.2175	0.9118	0.5511	0.8121	0.2894	0.5290	0.3749	0.8280	0.3587	0.8940	0.2892	0.6831	0.0429	0.1504	0.0841	0.4245	0.0932	0.3801	0.9483	0.9666	0.7327	0.8829	0.0963	0.3789	0.3308	0.7970	0.0646	0.2012
ACVR1B	0.0386	0.1365	0.0455	0.3874	0.5429	0.8121	0.9896	0.9896	0.1173	0.4781	0.0851	0.4751	0.0752	0.3624	0.0893	0.2254	0.5893	0.8078	0.4464	0.7784	0.1996	0.4069	0.0902	0.2516	0.0639	0.3789	0.6638	0.9402	0.2092	0.4330
ACVR2B	0.5201	0.7255	0.6239	0.9118	0.1401	0.5486	0.1394	0.2955	0.6368	0.9218	0.5199	0.8940	0.5539	0.8387	0.4803	0.6364	0.7436	0.8535	0.9546	0.9921	0.1479	0.3763	0.9167	0.9374	0.8773	0.9396	0.4819	0.8513	0.3462	0.5918
BMP4	0.3175	0.6301	0.5146	0.9118	0.2541	0.6668	0.3229	0.5520	0.7622	0.9218	0.7908	0.9426	0.8750	0.9481	0.8665	0.9322	0.8977	0.9150	0.9412	0.9921	0.1727	0.3866	0.8487	0.9180	0.9632	0.9632	0.4703	0.8513	0.6849	0.8320
CCND1	0.2296	0.5071	0.4210	0.9118	0.4188	0.7654	0.8940	0.9446	0.0454	0.2671	0.8538	0.9527	0.2563	0.6831	0.2936	0.4577	0.3165	0.6990	0.1007	0.3811	0.9386	0.9666	0.0322	0.1599	0.3276	0.6229	0.1517	0.5744	0.8987	0.9339
CCND2	0.0722	0.2189	0.8674	0.9863	0.2091	0.6517	0.0496	0.1314	0.6740	0.9218	0.4415	0.8940	0.9079	0.9481	0.1876	0.3908	0.5944	0.8078	0.2379	0.5730	0.0803	0.3040	0.3325	0.5875	0.3668	0.6481	0.7985	0.9402	0.2643	0.5003
CCND3	0.4373	0.6767	0.9708	0.9863	0.1449	0.5486	0.4098	0.6787	0.4853	0.9218	0.3256	0.8940	0.8191	0.9481	0.5902	0.7274	0.7290	0.8535	0.6068	0.8246	0.2933	0.5015	0.9567	0.9567	0.5404	0.7216	0.2654	0.7970	0.7193	0.8320
CDK4	0.1696	0.4281	0.5051	0.9118	0.2793	0.6668	0.0229	0.0715	0.5965	0.9218	0.0634	0.4199	0.1942	0.6432	0.0182	0.0805	0.0658	0.3874	0.0529	0.2337	0.0958	0.3359	0.5909	0.8610	0.0599	0.3789	0.9140	0.9498	0.0085	0.0528
CDK6	0.0004	0.0032	0.7787	0.9863	0.0124	0.0940	0.0017	0.0074	0.4418	0.9218	0.0986	0.4751	0.9367	0.9547	0.0284	0.1157	0.5240	0.7506	0.0090	0.1189	0.0011	0.0098	0.0631	0.2263	0.0920	0.3789	0.4576	0.8513	0.0820	0.2288
CDKN1A	0.0064	0.0339	0.5615	0.9118	0.4863	0.8054	0.0217	0.0715	0.0093	0.1910	0.8607	0.9527	0.2890	0.6831	0.2496	0.4267	0.0413	0.2739	0.1272	0.4496	0.0351	0.1565	0.0015	0.0520	0.5080	0.7216	0.1413	0.5744	0.3119	0.5510
CDKN2A	0.0000	0.0000	0.0000	0.0000	0.0000	0.0000	0.0000	0.0000	0.2831	0.7896	0.7871	0.9426	0.0129	0.0857	0.0463	0.1504	0.0020	0.0209	0.5659	0.8107	0.0000	0.0000	0.1683	0.4248	0.6379	0.7862	0.6020	0.9384	0.0000	0.0001
E2F3	0.3567	0.6301	0.8805	0.9863	0.3187	0.6668	0.0001	0.0004	0.0788	0.3797	0.4780	0.8940	0.0028	0.0249	0.0118	0.0697	0.0000	0.0003	0.6869	0.8307	0.2630	0.4806	0.8237	0.9095	0.7088	0.8348	0.0061	0.2472	0.0000	0.0004
ESR1	0.3565	0.6301	0.6565	0.9118	0.0838	0.4037	0.0276	0.0812	0.0183	0.1910	0.1201	0.5306	0.0065	0.0495	0.0534	0.1571	0.7013	0.8535	0.7984	0.9003	0.3655	0.5698	0.0202	0.1530	0.3046	0.6210	0.0767	0.4515	0.0314	0.1189
GLI1	0.0301	0.1141	0.5862	0.9118	0.9401	0.9684	0.0422	0.1176	0.0216	0.1910	0.4921	0.8940	0.8866	0.9481	0.0482	0.1504	0.0313	0.2367	0.0409	0.2337	0.1365	0.3763	0.0261	0.1599	0.1572	0.3967	0.1337	0.5744	0.0610	0.2012
GLI2	0.3267	0.6301	0.9344	0.9863	0.3967	0.7508	0.8620	0.9446	0.7611	0.9218	0.4253	0.8940	0.4035	0.6980	0.8224	0.9081	0.4979	0.7506	0.3996	0.7784	0.4485	0.6480	0.3250	0.5875	0.5735	0.7413	0.5678	0.9326	0.6620	0.8320
GLI3	0.9461	0.9881	0.9374	0.9863	0.9442	0.9684	0.5626	0.7483	0.9932	0.9932	0.9616	0.9616	0.3698	0.6980	0.5804	0.7274	0.4477	0.7506	0.9451	0.9921	0.7405	0.8766	0.8199	0.9095	0.9381	0.9562	0.8147	0.9402	0.3984	0.6088
HHIP	0.9695	0.9881	0.6709	0.9118	0.8318	0.9572	0.3179	0.5520	0.7952	0.9218	0.5692	0.8940	0.2650	0.6831	0.1674	0.3858	0.4607	0.7506	0.6677	0.8307	0.7517	0.8766	0.7040	0.8829	0.4499	0.7216	0.9510	0.9510	0.2354	0.4620
INHBA	0.0913	0.2548	0.0692	0.4584	0.7075	0.9094	0.2089	0.4061	0.0442	0.2671	0.0444	0.3918	0.8551	0.9481	0.4702	0.6364	0.1177	0.4456	0.4706	0.7784	0.2312	0.4377	0.2403	0.5306	0.1072	0.3789	0.0639	0.4231	0.4020	0.6088
INHBB	0.1165	0.3088	0.4276	0.9118	0.2945	0.6668	0.4844	0.7131	0.5276	0.9218	0.9549	0.9616	0.3946	0.6980	0.7990	0.9010	0.7739	0.8545	0.6897	0.8307	0.1598	0.3851	0.2368	0.5306	0.8374	0.9246	0.8035	0.9402	0.8282	0.9145
INHBC	0.6200	0.8015	0.5511	0.9118	0.7206	0.9094	0.8831	0.9446	0.3519	0.8280	0.2840	0.8855	0.5089	0.7933	0.4367	0.6091	0.8375	0.8877	0.8493	0.9377	0.8878	0.9411	0.4396	0.7232	0.4693	0.7216	0.4628	0.8513	0.7051	0.8320
MDM2	0.0000	0.0000	0.0001	0.0016	0.0000	0.0000	0.0000	0.0000	0.5109	0.9218	0.5543	0.8940	0.0008	0.0140	0.1640	0.3858	0.0138	0.1218	0.2344	0.5730	0.0000	0.0000	0.6011	0.8610	0.2303	0.5245	0.2053	0.6801	0.0002	0.0020
PTCH2	0.8414	0.9468	0.8296	0.9863	0.6999	0.9094	0.9009	0.9446	0.8875	0.9600	0.5912	0.8940	0.9051	0.9481	0.6863	0.8266	0.7405	0.8535	0.7311	0.8610	0.8158	0.8908	0.8850	0.9374	0.6644	0.8002	0.6749	0.9402	0.9621	0.9621
VDR	0.6073	0.8015	0.4180	0.9118	0.0617	0.3635	0.6091	0.7873	0.2671	0.7864	0.0292	0.3097	0.9123	0.9481	0.2072	0.4006	0.0998	0.4245	0.2827	0.6513	0.4163	0.6304	0.9197	0.9374	0.0866	0.3789	0.0565	0.4231	0.9430	0.9611

Validation set

Table 3: t-test p-value and adjusted p-value of miRNA qRT-PCR values of validation set samples

miRNAs	Muc vs SerHigh		Muc vs SerLow		Muc vs End		Muc vs Cc		SerHigh vs End		SerLow vs End		SerHigh vs Cc		SerLow vs Cc		End vs Cc		SerH vs SerL		Muc vs not Muc		SerHigh vs not Ser		SerLow vs not Ser		End vs not End		Cc vs not Cc	
	p	adj-p	p	adj-p	p	adj-p	p	adj-p	p	adj-p	p	adj-p	p	adj-p	p	adj-p	p	adj-p	p	adj-p	p	adj-p	p	adj-p	p	adj-p	p	adj-p	p	adj-p
hsa-let-7a-5p	0.0197	0.1303	0.1137	0.5020	0.0478	0.2531	0.0024	0.0141	0.3574	0.7894	0.0005	0.0235	0.2557	0.7395	0.0000	0.0001	0.0377	0.2687	0.0001	0.0043	0.0178	0.1176	0.1830	0.4612	0.0003	0.0091	0.7280	0.9754	0.0079	0.0702
hsa-let-7b-5p	0.5669	0.8915	0.3154	0.7640	0.9906	0.9906	0.2480	0.5986	0.5283	0.8207	0.3433	0.8677	0.0507	0.4182	0.0544	0.1921	0.1911	0.5627	0.4404	0.7258	0.9614	0.9799	0.3630	0.6634	0.2509	0.7000	0.9466	0.9822	0.0666	0.2940
hsa-let-7e-5p	0.7854	0.9258	0.2337	0.6880	0.6561	0.9496	0.4537	0.8292	0.9159	0.9518	0.2451	0.8677	0.6318	0.9058	0.5008	0.6806	0.6094	0.8281	0.3274	0.5984	0.5136	0.8758	0.7751	0.9237	0.2344	0.6903	0.8036	0.9822	0.6158	0.9136
hsa-miR-106b-5p	0.2688	0.7247	0.1031	0.5020	0.1080	0.4770	0.9268	0.9824	0.6669	0.9063	0.3905	0.8677	0.1436	0.6853	0.0227	0.1055	0.0507	0.2687	0.2580	0.5946	0.1443	0.5100	0.7743	0.9237	0.1627	0.6633	0.1475	0.6514	0.0900	0.3671
hsa-miR-125b-5p	0.9043	0.9530	0.0509	0.3854	0.8941	0.9496	0.6913	0.9548	0.7758	0.9214	0.0581	0.3423	0.5795	0.9058	0.1912	0.5534	0.7152	0.9476	0.0186	0.1647	0.7026	0.9034	0.5418	0.7797	0.0455	0.3012	0.6818	0.9754	0.7668	0.9744
hsa-miR-141-3p	0.7339	0.9258	0.3603	0.7652	0.6835	0.9496	0.5397	0.9228	0.9497	0.9586	0.4866	0.9055	0.3295	0.8732	0.2145	0.5534	0.2663	0.7078	0.4656	0.7258	0.7884	0.9062	0.8366	0.9237	0.3337	0.7313	0.6182	0.9637	0.2145	0.6065
hsa-miR-145-5p	0.2319	0.7247	0.1639	0.6611	0.8870	0.9496	0.4370	0.8273	0.2673	0.7894	0.1675	0.7399	0.0371	0.4182	0.2967	0.5534	0.3689	0.7720	0.0263	0.1700	0.9452	0.9799	0.0779	0.3666	0.0992	0.4780	0.8051	0.9822	0.2795	0.6440
hsa-miR-15a-5p	0.0228	0.1303	0.2276	0.6880	0.1416	0.5686	0.0038	0.0199	0.1736	0.7894	0.0197	0.2086	0.5757	0.9058	0.0004	0.0031	0.0461	0.2687	0.0045	0.0473	0.0458	0.2429	0.1030	0.3666	0.0079	0.0840	0.7361	0.9754	0.0210	0.1390
hsa-miR-16-5p	0.3168	0.7300	0.0906	0.5020	0.6012	0.9371	0.0688	0.2430	0.4495	0.8207	0.0288	0.2540	0.4492	0.9058	0.0036	0.0211	0.0903	0.3736	0.0289	0.1700	0.4184	0.8214	0.4096	0.6983	0.0142	0.1255	0.7223	0.9754	0.0532	0.2819
hsa-miR-17-5p	0.4846	0.8639	0.5343	0.8797	0.8228	0.9496	0.6752	0.9548	0.2908	0.7894	0.5960	0.9055	0.7007	0.9058	0.3216	0.5534	0.4931	0.7720	0.2958	0.5984	0.9372	0.9799	0.2828	0.5552	0.3900	0.7519	0.4639	0.8756	0.6342	0.9136
hsa-miR-192-5p	0.0000	0.0000	0.0002	0.0037	0.0000	0.0000	0.0000	0.0000	0.2547	0.7894	0.9131	0.9519	0.5797	0.9058	0.2970	0.5534	0.0916	0.3736	0.4608	0.7258	0.0000	0.0000	0.0535	0.3666	0.8705	0.9146	0.3375	0.8034	0.0123	0.0928
hsa-miR-194-5p	0.0000	0.0000	0.0020	0.0268	0.0000	0.0000	0.0003	0.0025	0.4169	0.7996	0.9340	0.9519	0.1693	0.6853	0.5861	0.7010	0.4313	0.7720	0.6884	0.8688	0.0000	0.0000	0.0372	0.3595	0.5218	0.7933	0.0626	0.4737	0.9598	0.9901
hsa-miR-195-5p	0.1869	0.6605	0.7878	0.9310	0.9062	0.9496	0.4353	0.8273	0.1365	0.7350	0.7439	0.9426	0.0453	0.4182	0.7296	0.7734	0.3242	0.7720	0.2490	0.5946	0.8036	0.9062	0.0407	0.3595	0.6763	0.8335	0.8374	0.9822	0.1634	0.5773
hsa-miR-19a-3p	0.5053	0.8639	0.0245	0.2162	0.0253	0.1914	0.9668	0.9854	0.1243	0.7350	0.3097	0.8677	0.5190	0.9058	0.0239	0.1055	0.0234	0.2071	0.0881	0.3887	0.1046	0.3961	0.4473	0.6983	0.0731	0.3877	0.0260	0.3450	0.0975	0.3691
hsa-miR-200c-3p	0.9882	0.9882	0.9567	0.9751	0.7418	0.9496	0.9119	0.9824	0.7014	0.9067	0.7702	0.9493	0.8941	0.9922	0.8893	0.8893	0.8646	0.9488	0.9611	0.9642	0.8568	0.9268	0.8084	0.9237	0.8565	0.9146	0.6630	0.9754	0.9904	0.9904
hsa-miR-20a-5p	0.8210	0.9258	0.7760	0.9310	0.3357	0.7413	0.7967	0.9548	0.4929	0.8207	0.3160	0.8677	0.9989	0.9989	0.6330	0.7010	0.4718	0.7720	0.6693	0.8652	0.5829	0.8827	0.8104	0.9237	0.4378	0.7933	0.2589	0.7830	0.8302	0.9778
hsa-miR-20b-5p	0.0538	0.2374	0.1812	0.6611	0.1772	0.6261	0.2437	0.5986	0.3531	0.7894	0.5886	0.9055	0.4693	0.9058	0.6473	0.7010	0.9671	0.9748	0.9642	0.9642	0.0822	0.3352	0.2175	0.4612	0.5538	0.7933	0.9727	0.9822	0.9714	0.9901
hsa-miR-214-3p	0.0006	0.0054	0.6141	0.8797	0.0344	0.2281	0.0012	0.0080	0.0811	0.7350	0.0402	0.3042	0.8047	0.9922	0.0001	0.0008	0.1454	0.4818	0.0002	0.0043	0.0028	0.0248	0.0173	0.2422	0.0174	0.1320	0.9480	0.9822	0.0662	0.2940
hsa-miR-26a-5p	0.0363	0.1749	0.5658	0.8797	0.2534	0.7325	0.0248	0.1010	0.1387	0.7350	0.8369	0.9519	0.8952	0.9922	0.2555	0.5534	0.1718	0.5356	0.3121	0.5984	0.0664	0.3201	0.0944	0.3666	0.6416	0.8264	0.4343	0.8728	0.1921	0.6065
hsa-miR-29a-3p	0.3008	0.7247	0.5843	0.8797	0.7398	0.9496	0.0198	0.0876	0.3571	0.7894	0.3929	0.8677	0.1167	0.6853	0.0466	0.1764	0.0082	0.1085	0.2170	0.5906	0.3316	0.7711	0.6346	0.8851	0.1990	0.6903	0.2422	0.7830	0.0047	0.0494
hsa-miR-29b-3p	0.2595	0.7247	0.0160	0.1700	0.5318	0.9371	0.2017	0.5626	0.4055	0.7996	0.0009	0.0235	0.9588	0.9922	0.0001	0.0009	0.3635	0.7720	0.0011	0.0144	0.5123	0.8758	0.2065	0.4612	0.0003	0.0091	0.7974	0.9822	0.2256	0.6065
hsa-miR-29c-3p	0.3348	0.7305	0.9540	0.9751	0.2870	0.7325	0.6128	0.9548	0.9112	0.9518	0.4573	0.9055	0.5983	0.9058	0.6402	0.7010	0.5521	0.8102	0.4297	0.7258	0.3061	0.7711	0.6905	0.9211	0.5293	0.7933	0.3423	0.8034	0.7674	0.9744
hsa-miR-30a-3p	0.2755	0.7247	0.5258	0.8797	0.5823	0.9371	0.0000	0.0000	0.0511	0.7350	0.7252	0.9426	0.0000	0.0000	0.0000	0.0000	0.0000	0.0000	0.1581	0.5235	0.1626	0.5386	0.0012	0.0618	0.8949	0.9146	0.1220	0.6464	0.0000	0.0000
hsa-miR-30a-5p	0.7476	0.9258	0.5667	0.8797	0.7989	0.9496	0.0000	0.0000	0.8720	0.9432	0.6157	0.9055	0.0000	0.0000	0.0000	0.0000	0.0000	0.0000	0.7437	0.8981	0.0780	0.3352	0.1203	0.3666	0.6342	0.8264	0.0029	0.0773	0.0000	0.0000
hsa-miR-324-5p	0.8086	0.9258	0.2956	0.7640	0.9138	0.9496	0.1532	0.4776	0.8258	0.9312	0.1591	0.7399	0.1865	0.6853	0.0418	0.1703	0.0574	0.2765	0.2003	0.5906	0.6907	0.9034	0.9711	0.9807	0.1200	0.5299	0.5893	0.9547	0.0320	0.1886
hsa-miR-34a-5p	0.7640	0.9258	0.9991	0.9991	0.2835	0.7325	0.4141	0.8273	0.5499	0.8207	0.3847	0.8677	0.6361	0.9058	0.4715	0.6576	0.9165	0.9647	0.8189	0.9388	0.3917	0.8214	0.7299	0.9211	0.6061	0.8264	0.4446	0.8728	0.6075	0.9136
hsa-miR-34b-5p	0.5021	0.8639	0.4902	0.8797	0.9538	0.9721	0.5670	0.9391	0.4225	0.7996	0.5262	0.9055	0.9535	0.9922	0.3192	0.5534	0.4953	0.7720	0.2707	0.5979	0.8436	0.9268	0.4221	0.6983	0.3587	0.7313	0.5866	0.9547	0.5226	0.9136
hsa-miR-424-5p	0.6492	0.9258	0.2590	0.7224	0.2595	0.7325	0.7447	0.9548	0.4913	0.8207	0.6205	0.9055	0.9113	0.9922	0.3678	0.5570	0.4431	0.7720	0.3833	0.6772	0.3346	0.7711	0.7130	0.9211	0.3973	0.7519	0.3334	0.8034	0.6189	0.9136
hsa-miR-449a	0.2929	0.7247	0.8080	0.9310	0.4624	0.8948	0.2617	0.6031	0.5608	0.8207	0.3885	0.8677	0.9012	0.9922	0.1921	0.5534	0.4897	0.7720	0.2340	0.5906	0.3106	0.7711	0.4548	0.6983	0.3369	0.7313	0.9698	0.9822	0.3996	0.8471
hsa-miR-96-5p	0.0004	0.0041	0.2223	0.6880	0.0001	0.0014	0.0051	0.0248	0.7997	0.9214	0.1323	0.7012	0.1975	0.6853	0.3237	0.5534	0.2671	0.7078	0.0953	0.3887	0.0000	0.0004	0.1032	0.3666	0.3406	0.7313	0.0209	0.3450	0.8897	0.9856

Table 4: t-test p-value and adjusted p-value of gene qRT-PCR values of validation set samples

miRNAs	Muc vs SerHigh		Muc vs SerLow		Muc vs End		Muc vs Cc		SerHigh vs End		SerLow vs End		SerHigh vs Cc		SerLow vs Cc		End vs Cc		SerH vs SerL		Muc vs not Muc		SerHigh vs not Ser		SerLow vs not Ser		End vs not End		Cc vs not Cc	
	p	adj-p	p	adj-p	p	adj-p	p	adj-p	p	adj-p	p	adj-p	p	adj-p	p	adj-p	p	adj-p	p	adj-p	p	adj-p	p	adj-p	p	adj-p	p	adj-p	p	adj-p
ACVR1	0.6707	0.9258	0.8009	0.9310	0.3243	0.7413	0.9918	0.9918	0.7243	0.9140	0.6739	0.9210	0.6976	0.9058	0.8502	0.8665	0.3708	0.7720	0.9133	0.9491	0.5453	0.8758	0.9402	0.9770	0.9146	0.9146	0.3515	0.8034	0.5103	0.9136
ACVR1B	0.1143	0.4327	0.3361	0.7652	0.4727	0.8948	0.7914	0.9548	0.2580	0.7894	0.6777	0.9210	0.0730	0.4838	0.2708	0.5534	0.3175	0.7720	0.7626	0.8981	0.3748	0.8214	0.0974	0.3666	0.6267	0.8264	0.9609	0.9822	0.1994	0.6065
ACVR2B	0.8795	0.9530	0.3920	0.7991	0.1502	0.5686	0.9507	0.9854	0.0746	0.7350	0.9265	0.9519	0.8124	0.9922	0.3218	0.5534	0.1289	0.4553	0.3138	0.5984	0.4041	0.8214	0.2018	0.4612	0.5074	0.7933	0.0551	0.4737	0.4351	0.8599
BMP4	0.7113	0.9258	0.7377	0.9310	0.7597	0.9496	0.7614	0.9548	0.4146	0.7996	0.8830	0.9519	0.8981	0.9922	0.4589	0.6573	0.4800	0.7720	0.5478	0.7641	0.9867	0.9867	0.4612	0.6983	0.6956	0.8379	0.4386	0.8728	0.6378	0.9136
CCND1	0.5975	0.8915	0.3171	0.7640	0.3704	0.7854	0.8311	0.9548	0.1223	0.7350	0.5832	0.9055	0.4598	0.9058	0.3579	0.5570	0.4784	0.7720	0.2258	0.5906	0.6360	0.9034	0.1405	0.3919	0.3175	0.7313	0.2233	0.7830	0.8250	0.9778
CCND2	0.8905	0.9530	0.6629	0.9106	0.3013	0.7325	0.1793	0.5279	0.3419	0.7894	0.7469	0.9426	0.2069	0.6853	0.5200	0.6890	0.5809	0.8102	0.7602	0.8981	0.3208	0.7711	0.3549	0.6634	0.8729	0.9146	0.4956	0.8756	0.2573	0.6440
CCND3	0.7154	0.9258	0.7033	0.9310	0.8552	0.9496	0.7158	0.9548	0.7797	0.9214	0.5898	0.9055	0.9516	0.9922	0.4247	0.6253	0.8247	0.9488	0.5316	0.7641	0.8015	0.9062	0.6985	0.9211	0.5429	0.7933	0.9822	0.9822	0.7906	0.9744
CDK4	0.0246	0.1303	0.6019	0.8797	0.0455	0.2531	0.1159	0.3841	0.6608	0.9063	0.0514	0.3408	0.5741	0.9058	0.0933	0.3089	0.8534	0.9488	0.0248	0.1700	0.0405	0.2382	0.2166	0.4612	0.0560	0.3296	0.2559	0.7830	0.7185	0.9744
CDK6	0.0056	0.0425	0.1871	0.6611	0.0083	0.0733	0.0333	0.1260	0.7991	0.9214	0.0016	0.0290	0.6094	0.9058	0.0022	0.0146	0.7788	0.9488	0.0002	0.0043	0.0085	0.0641	0.1622	0.4299	0.0023	0.0406	0.0728	0.4823	0.5551	0.9136
CDKN1A	0.6056	0.8915	0.0680	0.4504	0.3033	0.7325	0.2485	0.5986	0.6966	0.9067	0.0043	0.0569	0.4799	0.9058	0.0175	0.0930	0.5724	0.8102	0.0327	0.1735	0.4573	0.8656	0.9807	0.9807	0.0073	0.0840	0.3638	0.8034	0.2289	0.6065
CDKN2A	0.0000	0.0000	0.0000	0.0004	0.0000	0.0000	0.0000	0.0000	0.2628	0.7894	0.8882	0.9519	0.2651	0.7395	0.2664	0.5534	0.0196	0.2071	0.5999	0.7949	0.0000	0.0000	0.1084	0.3666	0.7452	0.8777	0.4942	0.8756	0.0014	0.0182
E2F3	0.4943	0.8639	0.9048	0.9642	0.8292	0.9496	0.0000	0.0000	0.3158	0.7894	0.9658	0.9658	0.0000	0.0000	0.0000	0.0000	0.0000	0.0000	0.5334	0.7641	0.1979	0.5827	0.0183	0.2422	0.5159	0.7933	0.0455	0.4737	0.0000	0.0000
ESR1	0.9517	0.9700	0.8446	0.9325	0.4956	0.9058	0.4172	0.8273	0.5125	0.8207	0.8000	0.9519	0.4401	0.9058	0.6481	0.7010	0.7728	0.9488	0.8785	0.9388	0.5378	0.8758	0.5443	0.7797	0.9104	0.9146	0.5944	0.9547	0.5342	0.9136
GLI1	0.3446	0.7305	0.3609	0.7652	0.8324	0.9496	0.7101	0.9548	0.3575	0.7894	0.2795	0.8677	0.6729	0.9058	0.3361	0.5566	0.7763	0.9488	0.1068	0.4043	0.7159	0.9034	0.2820	0.5552	0.2240	0.6903	0.8206	0.9822	0.7845	0.9744
GLI2	0.2803	0.7247	0.4730	0.8797	0.7671	0.9496	0.7003	0.9548	0.1908	0.7894	0.2304	0.8677	0.4650	0.9058	0.3102	0.5534	0.7975	0.9488	0.1489	0.5235	0.6024	0.8869	0.1245	0.3666	0.2234	0.6903	0.7235	0.9754	0.9113	0.9856
GLI3	0.5944	0.8915	0.8280	0.9325	0.5929	0.9371	0.3003	0.6631	0.8621	0.9432	0.4983	0.9055	0.5920	0.9058	0.2485	0.5534	0.4025	0.7720	0.5224	0.7641	0.4746	0.8674	0.8341	0.9237	0.4686	0.7933	0.9707	0.9822	0.3421	0.7556
HHIP	0.3847	0.7842	0.6116	0.8797	0.0911	0.4390	0.8648	0.9548	0.5457	0.8207	0.5287	0.9055	0.4223	0.9058	0.6373	0.7010	0.0998	0.3779	0.8636	0.9388	0.1950	0.5827	0.8698	0.9408	0.8915	0.9146	0.0860	0.5064	0.2708	0.6440
INHBA	0.9171	0.9530	0.7643	0.9310	0.3040	0.7325	0.7134	0.9548	0.2349	0.7894	0.3344	0.8677	0.6534	0.9058	0.5978	0.7010	0.5291	0.8012	0.8396	0.9388	0.5775	0.8827	0.4088	0.6983	0.4943	0.7933	0.1435	0.6514	0.9534	0.9901
INHBB	0.0785	0.3201	0.4672	0.8797	0.7881	0.9496	0.8502	0.9548	0.0101	0.5329	0.5457	0.9055	0.0552	0.4182	0.5934	0.7010	0.9748	0.9748	0.0599	0.2887	0.7834	0.9062	0.0039	0.1022	0.3333	0.7313	0.2348	0.7830	0.5572	0.9136
INHBC	0.8186	0.9258	0.1093	0.5020	0.7498	0.9496	0.8518	0.9548	0.9586	0.9586	0.2727	0.8677	0.9734	0.9922	0.3179	0.5534	0.9283	0.9647	0.3212	0.5984	0.6774	0.9034	0.9108	0.9654	0.2326	0.6903	0.9483	0.9822	0.8751	0.9856
MDM2	0.0000	0.0000	0.0000	0.0005	0.0000	0.0000	0.0000	0.0001	0.5729	0.8207	0.8412	0.9519	0.2565	0.7395	0.3630	0.5570	0.0468	0.2687	0.8856	0.9388	0.0000	0.0000	0.1231	0.3666	0.3553	0.7313	0.0002	0.0128	0.8971	0.9856
PTCH2	0.5566	0.8915	0.9096	0.9642	0.4193	0.8548	0.4734	0.8364	0.0935	0.7350	0.6321	0.9055	0.1839	0.6853	0.6194	0.7010	0.8772	0.9488	0.5712	0.7762	0.6728	0.9034	0.0979	0.3666	0.9043	0.9146	0.2659	0.7830	0.4381	0.8599
VDR	0.4853	0.8639	0.6701	0.9106	0.5593	0.9371	0.7445	0.9548	0.0940	0.7350	0.9047	0.9519	0.2022	0.6853	0.7860	0.8168	0.8260	0.9488	0.2252	0.5906	0.7857	0.9062	0.1083	0.3666	0.6549	0.8264	0.2884	0.8034	0.7781	0.9744

Concordant results training and validation set

Table 5: t-test p-value and adjusted p-value of qRT-PCR expression values in training set samples

	Training set																			
	Muc vs SerHigh		Muc vs SerLow		Muc vs End		Muc vs Cc		SerHigh vs End		SerLow vs End		SerHigh vs Cc		SerLow vs Cc		End vs Cc		SerH vs SerL	
	p	adj-p	p	adj-p	p	adj-p	p	adj-p	p	adj-p	p	adj-p	p	adj-p	p	adj-p	p	adj-p	p	adj-p
hsa-let-7a-5p	0.0197	0.1303	0.1137	0.5020	0.0478	0.2531	0.0024	0.0141	0.3574	0.7894	0.0005	0.0235	0.2557	0.7395	0.0000	0.0001	0.0377	0.2687	0.0001	0.0043
hsa-miR-192-5p	0.0000	0.0000	0.0002	0.0037	0.0000	0.0000	0.0000	0.0000	0.2547	0.7894	0.9131	0.9519	0.5797	0.9058	0.2970	0.5534	0.0916	0.3736	0.4608	0.7258
hsa-miR-194-5p	0.0000	0.0000	0.0020	0.0268	0.0000	0.0000	0.0003	0.0025	0.4169	0.7996	0.9340	0.9519	0.1693	0.6853	0.5861	0.7010	0.4313	0.7720	0.6884	0.8688
hsa-miR-214-3p	0.0006	0.0054	0.6141	0.8797	0.0344	0.2281	0.0012	0.0080	0.0811	0.7350	0.0402	0.3042	0.8047	0.9922	0.0001	0.0008	0.1454	0.4818	0.0002	0.0043
hsa-miR-26a-5p	0.0363	0.1749	0.5658	0.8797	0.2534	0.7325	0.0248	0.1010	0.1387	0.7350	0.8369	0.9519	0.8952	0.9922	0.2555	0.5534	0.1718	0.5356	0.3121	0.5984
hsa-miR-29a-3p	0.3008	0.7247	0.5843	0.8797	0.7398	0.9496	0.0198	0.0876	0.3571	0.7894	0.3929	0.8677	0.1167	0.6853	0.0466	0.1764	0.0082	0.1085	0.2170	0.5906
hsa-miR-29b-3p	0.2595	0.7247	0.0160	0.1700	0.5318	0.9371	0.2017	0.5626	0.4055	0.7996	0.0009	0.0235	0.9588	0.9922	0.0001	0.0009	0.3635	0.7720	0.0011	0.0144
hsa-miR-30a-3p	0.2755	0.7247	0.5258	0.8797	0.5823	0.9371	0.0000	0.0000	0.0511	0.7350	0.7252	0.9426	0.0000	0.0000	0.0000	0.0000	0.0000	0.0000	0.1581	0.5235
hsa-miR-30a-5p	0.7476	0.9258	0.5667	0.8797	0.7989	0.9496	0.0000	0.0000	0.8720	0.9432	0.6157	0.9055	0.0000	0.0000	0.0000	0.0000	0.0000	0.0000	0.7437	0.8981
hsa-miR-96-5p	0.0004	0.0041	0.2223	0.6880	0.0001	0.0014	0.0051	0.0248	0.7997	0.9214	0.1323	0.7012	0.1975	0.6853	0.3237	0.5534	0.2671	0.7078	0.0953	0.3887
CDK6	0.0056	0.0425	0.1871	0.6611	0.0083	0.0733	0.0333	0.1260	0.7991	0.9214	0.0016	0.0290	0.6094	0.9058	0.0022	0.0146	0.7788	0.9488	0.0002	0.0043
CDKN1A	0.6056	0.8915	0.0680	0.4504	0.3033	0.7325	0.2485	0.5986	0.6966	0.9067	0.0043	0.0569	0.4799	0.9058	0.0175	0.0930	0.5724	0.8102	0.0327	0.1735
CDKN2A	0.0000	0.0000	0.0000	0.0004	0.0000	0.0000	0.0000	0.0000	0.2628	0.7894	0.8882	0.9519	0.2651	0.7395	0.2664	0.5534	0.0196	0.2071	0.5999	0.7949
E2F3	0.4943	0.8639	0.9048	0.9642	0.8292	0.9496	0.0000	0.0000	0.3158	0.7894	0.9658	0.9658	0.0000	0.0000	0.0000	0.0000	0.0000	0.0000	0.5334	0.7641
MDM2	0.0000	0.0000	0.0000	0.0005	0.0000	0.0000	0.0000	0.0001	0.5729	0.8207	0.8412	0.9519	0.2565	0.7395	0.3630	0.5570	0.0468	0.2687	0.8856	0.9388

Table 6: t-test p-value and adjusted p-value of qRT-PCR expression values in validation set samples

	Validation set																			
	Muc vs SerHigh		Muc vs SerLow		Muc vs End		Muc vs Cc		SerHigh vs End		SerLow vs End		SerHigh vs Cc		SerLow vs Cc		End vs Cc		SerH vs SerL	
	p	adj-p	p	adj-p	p	adj-p	p	adj-p	p	adj-p	p	adj-p	p	adj-p	p	adj-p	p	adj-p	p	adj-p
hsa-let-7a-5p	0.0012	0.0091	0.9849	0.9863	0.0602	0.3635	0.0016	0.0074	0.0520	0.2758	0.1683	0.5946	0.4018	0.6980	0.0169	0.0805	0.1688	0.5215	0.0221	0.2328
hsa-miR-192-5p	0.0000	0.0000	0.0000	0.0000	0.0000	0.0000	0.0000	0.0000	0.6842	0.9218	0.8806	0.9527	0.1767	0.6247	0.7796	0.8982	0.6812	0.8535	0.3771	0.7724
hsa-miR-194-5p	0.0000	0.0000	0.0000	0.0000	0.0000	0.0000	0.0000	0.0000	0.8083	0.9218	0.7085	0.8940	0.1768	0.6247	0.9347	0.9527	0.5082	0.7506	0.4415	0.7784
hsa-miR-214-3p	0.0000	0.0005	0.6570	0.9118	0.0014	0.0153	0.0000	0.0001	0.3073	0.8143	0.0040	0.1054	0.8778	0.9481	0.0000	0.0001	0.2122	0.5367	0.0002	0.0096
hsa-miR-26a-5p	0.0072	0.0346	0.9291	0.9863	0.0790	0.4037	0.0033	0.0135	0.3287	0.8280	0.1551	0.5874	0.9999	0.9999	0.0095	0.0630	0.2958	0.6990	0.0283	0.2328
hsa-miR-29a-3p	0.1899	0.4575	0.0512	0.3874	0.3262	0.6668	0.0957	0.2205	0.7376	0.9218	0.0132	0.1754	0.7564	0.9481	0.0025	0.0187	0.5136	0.7506	0.0070	0.1189
hsa-miR-29b-3p	0.0044	0.0260	0.1692	0.9118	0.0031	0.0274	0.0003	0.0016	0.9426	0.9796	0.0024	0.1054	0.4083	0.6980	0.0007	0.0097	0.4482	0.7506	0.0035	0.0917
hsa-miR-30a-3p	0.8097	0.9331	0.5548	0.9118	0.8213	0.9572	0.0000	0.0000	0.9633	0.9818	0.7056	0.8940	0.0000	0.0000	0.0000	0.0000	0.0000	0.0000	0.4771	0.7784
hsa-miR-30a-5p	0.9686	0.9881	0.5980	0.9118	0.7880	0.9492	0.0000	0.0000	0.7934	0.9218	0.6702	0.8940	0.0000	0.0000	0.0000	0.0000	0.0000	0.0000	0.5494	0.8089
hsa-miR-96-5p	0.4640	0.6767	0.9863	0.9863	0.5985	0.8572	0.0003	0.0018	0.7916	0.9218	0.6623	0.8940	0.0011	0.0140	0.0020	0.0176	0.0004	0.0058	0.5140	0.7784
CDK6	0.0004	0.0032	0.7787	0.9863	0.0124	0.0940	0.0017	0.0074	0.4418	0.9218	0.0986	0.4751	0.9367	0.9547	0.0284	0.1157	0.5240	0.7506	0.0090	0.1189
CDKN1A	0.0064	0.0339	0.5615	0.9118	0.4863	0.8054	0.0217	0.0715	0.0093	0.1910	0.8607	0.9527	0.2890	0.6831	0.2496	0.4267	0.0413	0.2739	0.1272	0.4496
CDKN2A	0.0000	0.0000	0.0000	0.0000	0.0000	0.0000	0.0000	0.0000	0.2831	0.7896	0.7871	0.9426	0.0129	0.0857	0.0463	0.1504	0.0020	0.0209	0.5659	0.8107
E2F3	0.3567	0.6301	0.8805	0.9863	0.3187	0.6668	0.0001	0.0004	0.0788	0.3797	0.4780	0.8940	0.0028	0.0249	0.0118	0.0697	0.0000	0.0003	0.6869	0.8307
MDM2	0.0000	0.0000	0.0001	0.0016	0.0000	0.0000	0.0000	0.0000	0.5109	0.9218	0.5543	0.8940	0.0008	0.0140	0.1640	0.3858	0.0138	0.1218	0.2344	0.5730

Boxplots of qRT-PCR expression values across histotypes

Training set

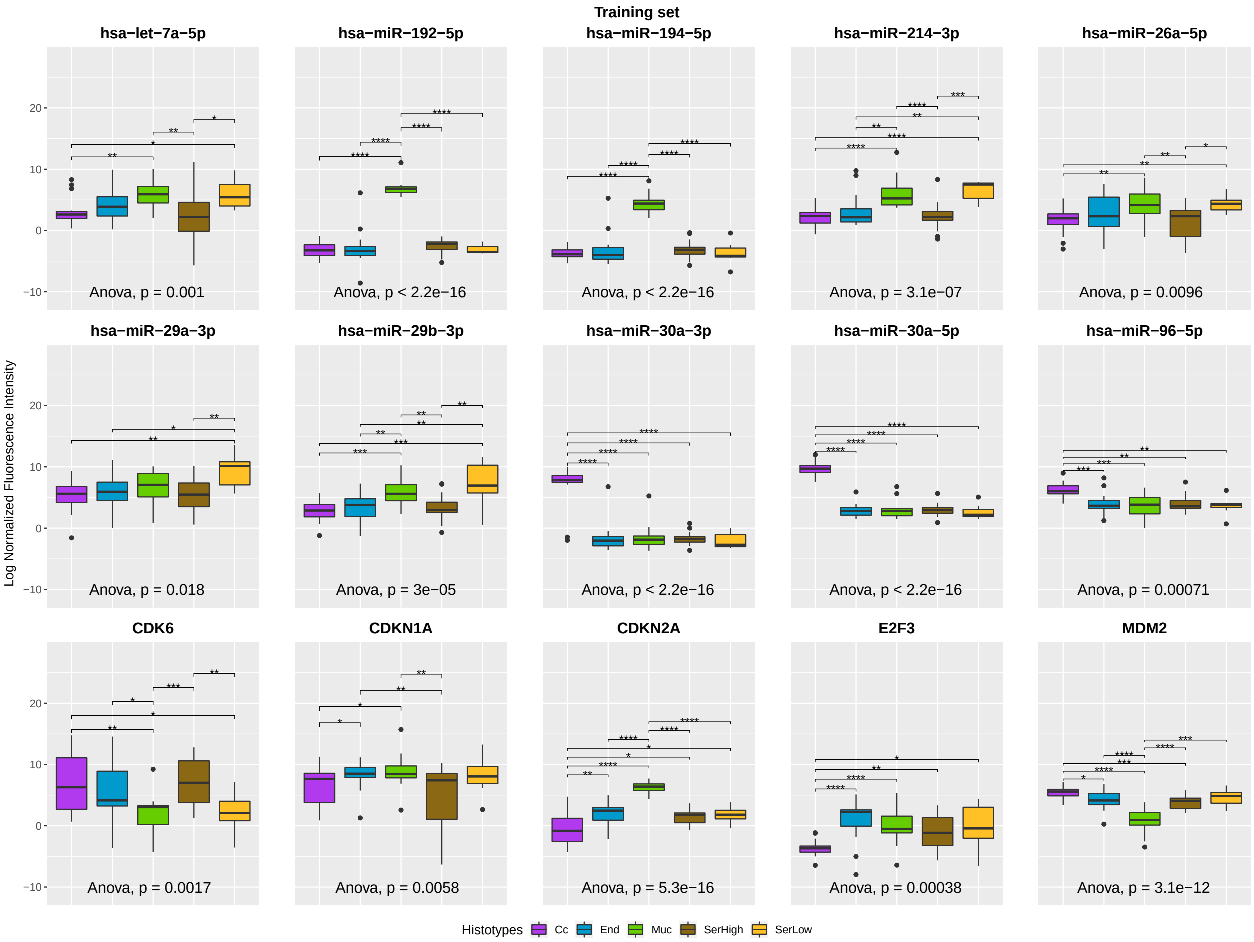

Tables of average values of qRT-PCR expression across histotypes

Training set

Table 7: Means of qRT-PCR expression values across histotypes in training set patients

	Muc	SerHigh	SerLow	End	Cc	Not Muc	Not SerHigh	Not SerLow	Not End	not Cc
ACVR1	8.062	8.232	6.314	7.469	9.237	8.010	7.961	8.198	8.210	7.718
ACVR1B	8.179	6.841	6.204	7.784	8.189	7.435	7.820	7.743	7.540	7.444
ACVR2B	7.456	8.278	8.182	8.745	8.945	8.598	8.361	8.359	8.208	8.182
BMP4	4.757	5.687	5.615	5.970	5.832	5.811	5.536	5.564	5.430	5.502
CCND1	9.941	11.081	9.123	9.291	10.050	9.993	9.664	10.068	10.211	9.963
CCND2	7.957	9.122	8.075	8.815	9.202	8.921	8.596	8.781	8.681	8.583
CCND3	8.319	8.736	8.342	9.173	8.908	8.877	8.764	8.801	8.617	8.717
CDK4	4.144	6.232	2.727	5.623	8.271	6.173	5.571	6.023	5.751	5.039
CDK6	1.730	7.106	2.192	5.865	6.981	6.076	4.567	5.434	4.881	4.602
CDKN1A	8.927	4.819	8.158	8.353	6.431	6.790	7.974	7.178	6.907	7.491
CDKN2A	6.289	1.445	1.802	1.994	-0.475	1.143	2.539	2.344	2.395	3.033
E2F3	-0.079	-1.013	-0.324	1.000	-3.646	-1.095	-0.805	-0.892	-1.435	-0.039
ESR1	3.191	2.173	2.584	5.060	5.484	4.049	4.343	3.987	3.456	3.424
GLI1	10.382	7.757	11.020	10.315	7.946	9.019	9.775	9.152	8.994	9.692
GLI2	1.715	3.284	1.543	2.877	1.988	2.595	2.143	2.485	2.238	2.507
GLI3	-0.303	-0.400	-0.474	-0.408	-1.181	-0.631	-0.614	-0.576	-0.620	-0.387
HHIP	6.924	6.896	6.508	7.086	7.596	7.101	7.109	7.118	7.053	6.919
hsa-let-7a-5p	5.913	2.157	5.933	4.304	3.091	3.550	4.632	3.890	4.003	4.342
hsa-let-7b-5p	3.422	3.051	2.675	2.601	2.002	2.577	2.684	2.775	2.821	2.970
hsa-let-7e-5p	7.650	6.919	6.920	7.528	6.504	7.003	7.213	7.170	7.021	7.319
hsa-miR-106b-5p	3.093	3.121	3.476	3.065	3.614	3.282	3.271	3.214	3.298	3.137
hsa-miR-125b-5p	4.650	5.080	5.612	3.835	3.011	4.181	4.057	4.151	4.436	4.626
hsa-miR-141-3p	6.142	5.247	7.270	7.610	6.243	6.518	6.776	6.349	6.042	6.485
hsa-miR-145-5p	5.027	5.212	6.363	4.431	4.590	4.929	4.875	4.807	5.124	5.047
hsa-miR-15a-5p	2.954	2.168	3.824	2.418	2.289	2.478	2.704	2.458	2.640	2.663
hsa-miR-16-5p	4.964	2.850	4.919	5.015	3.830	4.059	4.668	4.195	4.010	4.376
hsa-miR-17-5p	2.167	3.967	1.658	2.671	3.222	3.071	2.544	2.991	2.932	2.777
hsa-miR-192-5p	6.849	-2.607	-3.070	-2.907	-3.220	-2.925	-0.200	-0.502	-0.016	-0.077
hsa-miR-194-5p	4.484	-3.110	-3.672	-3.278	-3.724	-3.397	-1.209	-1.427	-1.086	-1.077
hsa-miR-195-5p	7.335	4.014	7.800	5.540	3.819	4.902	5.859	5.207	5.415	5.880
hsa-miR-19a-3p	2.181	1.948	1.422	2.147	2.858	2.178	2.247	2.263	2.189	2.009
hsa-miR-200c-3p	5.831	5.836	6.279	6.101	5.759	5.953	5.951	5.890	5.867	5.970
hsa-miR-20a-5p	11.025	11.520	10.696	11.130	11.373	11.257	11.114	11.256	11.230	11.160
hsa-miR-20b-5p	3.039	2.711	3.297	2.507	3.192	2.848	2.936	2.844	3.021	2.801
hsa-miR-214-3p	6.005	2.232	6.461	3.076	2.126	2.977	4.064	3.370	3.847	4.062
hsa-miR-26a-5p	4.236	1.666	4.323	2.617	1.666	2.288	3.028	2.561	2.759	3.005
hsa-miR-29a-3p	6.773	5.499	9.296	5.828	5.203	5.975	6.342	5.835	6.262	6.407
hsa-miR-29b-3p	5.583	3.354	7.305	3.302	2.784	3.651	4.294	3.757	4.344	4.430
hsa-miR-29c-3p	4.573	4.073	4.661	3.814	2.682	3.680	3.826	3.802	3.904	4.208
hsa-miR-30a-3p	-1.525	-1.660	-2.033	-1.687	6.932	0.617	0.656	0.358	0.746	-1.674
hsa-miR-30a-5p	2.977	2.960	2.655	2.867	9.724	4.728	4.733	4.507	4.826	2.900
hsa-miR-324-5p	4.625	2.128	2.777	3.914	4.343	3.393	4.151	3.746	3.603	3.494
hsa-miR-34a-5p	4.005	3.649	5.898	5.202	4.347	4.605	4.708	4.326	4.227	4.504
hsa-miR-34b-5p	1.441	2.009	2.014	1.924	1.281	1.785	1.621	1.677	1.636	1.822
hsa-miR-424-5p	10.062	9.527	11.074	9.856	9.873	9.910	10.064	9.830	9.973	9.963
hsa-miR-449a	3.834	1.236	3.127	3.549	3.203	2.739	3.487	2.969	2.795	2.925
hsa-miR-96-5p	3.639	4.090	3.625	3.942	6.186	4.464	4.298	4.331	4.380	3.845
INHBA	7.123	5.441	4.356	7.488	5.654	6.029	6.514	6.468	5.869	6.439
INHBB	-1.905	-3.517	-3.007	-2.936	-2.656	-3.036	-2.583	-2.773	-2.747	-2.832
INHBC	1.327	0.854	0.623	1.606	1.457	1.232	1.369	1.317	1.136	1.199
MDM2	0.762	3.888	4.590	4.194	5.370	4.471	3.571	3.546	3.458	3.181
PTCH2	7.129	6.971	7.338	6.868	7.052	7.003	7.049	7.001	7.086	7.026
VDR	3.386	3.927	2.169	4.908	3.823	4.006	3.850	4.040	3.521	3.879

Validation set

Table 8: Means of qRT-PCR expression values across histotypes in validation set patients

	Muc	SerHigh	SerLow	End	Cc	Not Muc	Not SerHigh	Not SerLow	Not End	not Cc
ACVR1	8.854	9.148	9.041	9.324	8.847	9.168	9.113	9.125	8.972	9.175
ACVR1B	7.317	8.407	8.112	7.744	7.150	7.809	7.569	7.707	7.724	7.847
ACVR2B	5.056	4.800	7.023	6.868	5.154	6.055	6.176	5.830	5.204	6.054
BMP4	7.467	7.156	7.806	7.671	7.249	7.477	7.554	7.454	7.333	7.521
CCND1	6.330	6.934	4.674	5.493	6.125	5.896	5.722	6.046	6.303	5.930
CCND2	9.017	8.896	8.542	8.145	7.668	8.249	8.241	8.356	8.530	8.510
CCND3	9.068	8.722	9.509	8.929	8.773	8.896	8.969	8.883	8.918	8.952
CDK4	5.524	8.576	4.409	8.096	7.873	7.899	7.279	7.740	7.131	7.468
CDK6	5.130	8.737	2.854	8.465	8.136	8.056	7.323	7.917	6.995	7.497
CDKN1A	8.432	7.946	10.763	7.669	7.239	7.872	7.963	7.773	8.172	8.109
CDKN2A	5.759	0.616	1.227	1.370	-0.563	0.815	1.851	1.639	1.791	2.033
E2F3	-0.213	0.286	-0.310	-0.349	-4.678	-1.094	-1.234	-0.992	-1.373	-0.175
ESR1	7.024	6.949	6.701	6.359	6.104	6.475	6.462	6.550	6.706	6.647
GLI1	10.794	9.985	11.824	10.630	10.397	10.519	10.702	10.476	10.507	10.592
GLI2	-1.060	0.638	-2.459	-0.745	-0.478	-0.487	-0.882	-0.451	-0.458	-0.596
GLI3	-0.893	-0.441	-1.188	-0.541	-0.077	-0.465	-0.554	-0.494	-0.524	-0.624
HHIP	7.273	7.782	7.652	8.035	7.364	7.815	7.720	7.738	7.513	7.807
hsa-let-7a-5p	7.843	4.819	10.858	5.652	3.652	5.436	6.047	5.474	5.912	6.237
hsa-let-7b-5p	4.201	4.599	5.196	4.192	3.253	4.170	4.072	4.107	4.161	4.364
hsa-let-7e-5p	7.808	8.002	9.052	8.059	8.342	8.173	8.146	8.057	8.160	8.072
hsa-miR-106b-5p	3.585	4.590	5.816	4.883	3.503	4.603	4.409	4.355	4.140	4.639
hsa-miR-125b-5p	4.678	4.569	7.362	4.799	5.154	5.000	5.042	4.793	5.056	4.908
hsa-miR-141-3p	7.924	8.302	9.517	8.363	7.158	8.193	8.115	8.063	7.999	8.353
hsa-miR-145-5p	6.013	5.399	7.058	5.938	6.387	5.981	6.131	5.917	6.021	5.906
hsa-miR-15a-5p	4.446	2.438	5.794	3.359	2.008	3.054	3.460	3.092	3.189	3.521
hsa-miR-16-5p	6.335	5.594	7.920	6.027	5.048	5.866	6.025	5.809	5.876	6.120
hsa-miR-17-5p	3.562	4.078	2.891	3.419	3.825	3.609	3.492	3.649	3.735	3.555
hsa-miR-192-5p	7.935	0.871	2.144	1.967	0.205	1.367	2.803	2.435	2.747	2.866
hsa-miR-194-5p	6.133	0.847	1.388	1.496	2.176	1.471	2.548	2.266	2.724	2.220
hsa-miR-195-5p	7.179	5.753	7.500	7.074	7.945	6.968	7.309	6.967	6.947	6.809
hsa-miR-19a-3p	2.836	3.392	5.473	4.425	2.869	3.937	3.860	3.651	3.313	3.951
hsa-miR-200c-3p	8.603	8.622	8.714	8.255	8.446	8.426	8.407	8.440	8.591	8.460
hsa-miR-20a-5p	11.077	11.375	10.477	12.008	11.377	11.625	11.587	11.615	11.205	11.579
hsa-miR-20b-5p	2.520	3.886	3.843	3.354	3.379	3.517	3.236	3.331	3.368	3.359
hsa-miR-214-3p	7.774	4.588	8.469	5.883	4.742	5.547	6.230	5.737	5.919	6.126
hsa-miR-26a-5p	5.574	3.248	4.821	4.553	3.384	4.025	4.522	4.228	4.065	4.446
hsa-miR-29a-3p	7.144	6.200	7.942	6.878	4.670	6.359	6.547	6.383	6.189	6.843
hsa-miR-29b-3p	6.880	5.625	10.667	6.312	5.576	6.319	6.598	6.128	6.472	6.572
hsa-miR-29c-3p	8.492	7.269	8.611	7.147	7.837	7.407	7.658	7.523	7.895	7.529
hsa-miR-30a-3p	3.460	2.478	4.274	3.878	9.114	4.617	4.912	4.443	4.828	3.496
hsa-miR-30a-5p	5.146	5.371	5.681	5.286	10.339	6.336	6.337	6.176	6.761	5.308
hsa-miR-324-5p	3.704	3.432	5.319	3.609	1.921	3.356	3.404	3.285	3.265	3.710
hsa-miR-34a-5p	5.809	5.523	5.810	5.180	5.127	5.295	5.336	5.345	5.514	5.423
hsa-miR-34b-5p	2.716	2.378	3.224	2.744	2.409	2.627	2.705	2.602	2.565	2.687
hsa-miR-424-5p	11.150	11.515	12.481	11.962	11.427	11.788	11.735	11.640	11.495	11.744
hsa-miR-449a	3.009	2.182	3.301	2.511	2.103	2.410	2.580	2.449	2.494	2.582
hsa-miR-96-5p	3.146	5.856	4.314	5.704	5.052	5.502	4.950	5.172	4.697	5.132
INHBA	7.526	7.431	7.149	8.364	7.850	7.953	8.002	7.936	7.544	7.894
INHBB	-1.933	-0.004	-2.884	-2.159	-2.132	-1.701	-2.165	-1.662	-1.431	-1.656
INHBC	-0.109	-0.291	-1.471	-0.327	-0.261	-0.388	-0.359	-0.272	-0.359	-0.362
MDM2	-0.345	3.863	3.981	4.113	3.117	3.847	3.013	3.129	2.514	3.193
PTCH2	6.582	7.002	6.487	6.165	6.089	6.369	6.253	6.396	6.573	6.465
VDR	4.094	4.607	3.617	3.718	3.841	3.943	3.808	3.989	4.146	3.991
