# Supplementary material for: Transcriptional Characterization of Stage I Epithelial Ovarian Cancer: A Multicentric Study
Source: Cells. 2019 Dec 1;8(12):1554. doi: 10.3390/cells8121554 (PMC6952972; doi:10.3390/cells8121554)
Supplement: Supplementary file 1 [file cells-08-01554-s001.zip › SuppMat6.pdf]

Supplementary File 6: Gene Ontology enrichment analyses across histoptypes

Contents

Biological Process GO analysis . . . 1
Molecular Function GO analysis . . . 4
Summary of GO analyses . . . 5

We used Empirical Bayes linear model to identify differentially expressed genes ($ajusted\ p-value < 0.05$) across histotypes. On the lists of differentially expressed genes we perform GO enrichment analyses ($adj.p - value < 0.05$) using Biological Processes and Molecular Function GO categories. The table with the numbers of differentially expressed genes and the complete list of the significant GO terms are reported below.

Biological Process GO analysis

Table 1: Significant biological processes (p.adj<0.05) for differentially expressed genes between Cc and End subtypes

	Description	GeneRatio	qvalue
GO:0042219	cellular modified amino acid catabolic process	4/61	0.0008458
GO:0044273	sulfur compound catabolic process	5/61	0.0008458
GO:0043171	peptide catabolic process	3/61	0.0322215
GO:0006691	leukotriene metabolic process	3/61	0.0322215

Table 2: Significant biological processes (p.adj<0.05) for differentially expressed genes between Cc and SerH subtypes

	Description	GeneRatio	qvalue
GO:0043062	extracellular structure organization	13/79	0.0000779
GO:0042219	cellular modified amino acid catabolic process	4/79	0.0009835
GO:0006790	sulfur compound metabolic process	10/79	0.0073610
GO:0030203	glycosaminoglycan metabolic process	6/79	0.0148850
GO:0006022	aminoglycan metabolic process	6/79	0.0148850
GO:0001704	formation of primary germ layer	5/79	0.0148850
GO:0006024	glycosaminoglycan biosynthetic process	5/79	0.0148850
GO:0006749	glutathione metabolic process	4/79	0.0148850
GO:0043171	peptide catabolic process	3/79	0.0148850
GO:0006023	aminoglycan biosynthetic process	5/79	0.0148850
GO:0001944	vasculature development	12/79	0.0158292
GO:0006691	leukotriene metabolic process	3/79	0.0158292
GO:0072358	cardiovascular system development	12/79	0.0158292
GO:0043410	positive regulation of MAPK cascade	9/79	0.0232866
GO:0035295	tube development	14/79	0.0232866
GO:0045445	myoblast differentiation	4/79	0.0232866
GO:0030206	chondroitin sulfate biosynthetic process	3/79	0.0232866
GO:0001503	ossification	8/79	0.0232866
GO:0044262	cellular carbohydrate metabolic process	7/79	0.0233773
GO:0048646	anatomical structure formation involved in morphogenesis	14/79	0.0234317
GO:0050650	chondroitin sulfate proteoglycan biosynthetic process	3/79	0.0247372
GO:0001649	osteoblast differentiation	6/79	0.0269180
GO:0007492	endoderm development	4/79	0.0280152
GO:0030334	regulation of cell migration	12/79	0.0341758

Table 3: Significant biological processes (p.adj<0.05) for differentially expressed genes between End and SerH subtypes

	Description	GeneRatio	qvalue
GO:0030111	regulation of Wnt signaling pathway	6/16	0.0005466
GO:0090101	negative regulation of transmembrane receptor protein serine/threonine kinase signaling pathway	4/16	0.0005466
GO:0090288	negative regulation of cellular response to growth factor stimulus	4/16	0.0009233
GO:0198738	cell-cell signaling by wnt	6/16	0.0009233
GO:1905114	cell surface receptor signaling pathway involved in cell-cell signaling	6/16	0.0013463
GO:0007179	transforming growth factor beta receptor signaling pathway	4/16	0.0016501
GO:0010771	negative regulation of cell morphogenesis involved in differentiation	3/16	0.0021217
GO:0071559	response to transforming growth factor beta	4/16	0.0024966
GO:0007413	axonal fasciculation	2/16	0.0052757
GO:0106030	neuron projection fasciculation	2/16	0.0052757
GO:0010977	negative regulation of neuron projection development	3/16	0.0057980
GO:0031345	negative regulation of cell projection organization	3/16	0.0081075
GO:0045668	negative regulation of osteoblast differentiation	2/16	0.0119682
GO:0001649	osteoblast differentiation	3/16	0.0122123
GO:0010718	positive regulation of epithelial to mesenchymal transition	2/16	0.0127060
GO:0051093	negative regulation of developmental process	5/16	0.0137094
GO:1902905	positive regulation of supramolecular fiber organization	3/16	0.0164806
GO:0071242	cellular response to ammonium ion	2/16	0.0164849
GO:0009887	animal organ morphogenesis	5/16	0.0164849
GO:0051495	positive regulation of cytoskeleton organization	3/16	0.0178034
GO:0030279	negative regulation of ossification	2/16	0.0179071
GO:0048646	anatomical structure formation involved in morphogenesis	5/16	0.0212569
GO:0009792	embryo development ending in birth or egg hatching	4/16	0.0212569
GO:0048640	negative regulation of developmental growth	2/16	0.0238615
GO:0007420	brain development	4/16	0.0246835
GO:0120035	regulation of plasma membrane bounded cell projection organization	4/16	0.0251142
GO:0061387	regulation of extent of cell growth	2/16	0.0273063
GO:0060322	head development	4/16	0.0274911
GO:0008037	cell recognition	2/16	0.0299299
GO:0032956	regulation of actin cytoskeleton organization	3/16	0.0320942
GO:0030509	BMP signaling pathway	2/16	0.0320942

Table 4: Significant biological processes (p.adj<0.05) for differentially expressed genes between Muc and SerL subtypes

	Description	GeneRatio	qvalue
GO:0035082	axoneme assembly	3/24	0.0081197
GO:0001578	microtubule bundle formation	3/24	0.0351059

Table 5: Significant biological processes (p.adj<0.05) for differentially expressed genes between SerH and SerL subtypes

	Description	GeneRatio	qvalue
GO:0051301	cell division	26/64	0.0000000
GO:0000280	nuclear division	16/64	0.0000000
GO:0140014	mitotic nuclear division	14/64	0.0000000
GO:0000819	sister chromatid segregation	10/64	0.0000012
GO:0090068	positive regulation of cell cycle process	11/64	0.0000055
GO:0044772	mitotic cell cycle phase transition	15/64	0.0000229
GO:0051983	regulation of chromosome segregation	7/64	0.0000470
GO:0007088	regulation of mitotic nuclear division	8/64	0.0000913
GO:0007051	spindle organization	7/64	0.0005417
GO:0071103	DNA conformation change	9/64	0.0005876
GO:0051310	metaphase plate congression	5/64	0.0008796
GO:0050000	chromosome localization	5/64	0.0017782
GO:0006281	DNA repair	11/64	0.0027198
GO:0006260	DNA replication	7/64	0.0132193
GO:0033045	regulation of sister chromatid segregation	4/64	0.0161808
GO:0051321	meiotic cell cycle	5/64	0.0181788
GO:0006974	cellular response to DNA damage stimulus	12/64	0.0199030
GO:0007093	mitotic cell cycle checkpoint	5/64	0.0226522
GO:1903524	positive regulation of blood circulation	3/64	0.0263921
GO:0007405	neuroblast proliferation	3/64	0.0288985
GO:0140013	meiotic nuclear division	4/64	0.0288985
GO:0031145	anaphase-promoting complex-dependent catabolic process	4/64	0.0347405
GO:0034080	CENP-A containing nucleosome assembly	3/64	0.0381363
GO:0061641	CENP-A containing chromatin organization	3/64	0.0381363
GO:0061351	neural precursor cell proliferation	4/64	0.0405630
GO:0051781	positive regulation of cell division	3/64	0.0446352

Molecular Function GO analysis

Table 6: Significant molecular processes (p.adj<0.05) for differentially expressed genes between Cc and End subtypes

	Description	GeneRatio	qvalue
GO:0008242	omega peptidase activity	3/60	0.0112791

Table 7: Significant molecular processes (p.adj<0.05) for differentially expressed genes between Cc and SerH subtypes

	Description	GeneRatio	qvalue
GO:0008242	omega peptidase activity	3/77	0.0272821

Table 8: Significant molecular processes (p.adj<0.05) for differentially expressed genes between End and Muc subtypes

	Description	GeneRatio	qvalue
GO:0048018	receptor ligand activity	2/5	0.0064343
GO:0030545	receptor regulator activity	2/5	0.0064343
GO:0042056	chemoattractant activity	1/5	0.0166797
GO:0042813	Wnt-activated receptor activity	1/5	0.0166797

Table 9: Significant molecular processes (p.adj<0.05) for differentially expressed genes between End and SerH subtypes

	Description	GeneRatio	qvalue
GO:0005109	frizzled binding	2/17	0.0129697
GO:0017147	Wnt-protein binding	2/17	0.0130445

Summary of GO analyses

Table 10: Number of differentially expressed gene across subtypes along with the number of signficantly enriched GO categories (Biological Processes and Molecular functions)

	N..Diff..Expr..genes	N..Biological.Processes	N..Molecular.Function
Cc - End	71	4	1
Cc - Muc	50	0	0
Cc - SerH	85	24	1
Cc - SerL	91	0	0
End - Muc	6	0	4
End - SerH	17	31	2
End - SerL	20	0	0
Muc - SerH	26	0	0
Muc - SerL	27	2	0
SerH - SerL	65	26	0
